# Adherence to Technology-Mediated Insomnia Treatment: A Meta-Analysis, Interviews, and Focus Groups

**DOI:** 10.2196/jmir.4115

**Published:** 2015-09-04

**Authors:** Corine Horsch, Jaap Lancee, Robbert Jan Beun, Mark A Neerincx, Willem-Paul Brinkman

**Affiliations:** ^1^ Interactive Intelligence Delft University of Technology Delft Netherlands; ^2^ Department of Clinical Psychology University of Amsterdam Amsterdam Netherlands; ^3^ Interaction Technology Utrecht University Utrecht Netherlands

**Keywords:** sleep initiation and maintenance disorders, patient compliance, meta-analysis, interview, focus groups, mobile apps, user-computer interface

## Abstract

**Background:**

Several technologies have been proposed to support the reduction of insomnia complaints. A user-centered assessment of these technologies could provide insight into underlying factors related to treatment adherence.

**Objective:**

Gaining insight into adherence to technology-mediated insomnia treatment as a solid base for improving those adherence rates by applying adherence-enhancing strategies.

**Methods:**

Adherence to technology-mediated sleep products was studied in three ways. First, a meta-analysis was performed to investigate adherence rates in technology-mediated insomnia therapy. Several databases were queried for technology-mediated insomnia treatments. After inclusion and exclusion steps, data from 18 studies were retrieved and aggregated to find an average adherence rate. Next, 15 semistructured interviews about sleep-support technologies were conducted to investigate perceived adherence. Lastly, several scenarios were written about the usage of a virtual sleep coach that could support adherence rates. The scenarios were discussed in six different focus groups consisting of potential users (n=15), sleep experts (n=7), and coaches (n=9).

**Results:**

From the meta-analysis, average treatment adherence appeared to be approximately 52% (95% CI 43%-61%) for technology-mediated insomnia treatments. This means that, on average, half of the treatment exercises were not executed, suggesting there is a substantial need for adherence and room for improvement in this area. However, the users in the interviews believed they adhered quite well to their sleep products. Users mentioned relying on personal commitment (ie, willpower) for therapy adherence. Participants of the focus groups reconfirmed their belief in the effectiveness of personal commitment, which they regarded as more effective than adherence-enhancing strategies.

**Conclusions:**

Although adherence rates for insomnia interventions indicate extensive room for improvement, users might not consider adherence to be a problem; they believe willpower to be an effective adherence strategy. A virtual coach should be able to cope with this “adherence bias” and persuade users to accept adherence-enhancing strategies, such as reminders, compliments, and community building.

## Introduction

### Overview

People who suffer from insomnia have difficulties with initiating sleep, maintaining sleep, or early-morning awakenings, and this sleep disturbance significantly impairs their daily functioning [1]. Having insomnia may lead to personal suffering, such as feeling tired after a night’s sleep, reduced quality of life, and vulnerability to depression [2,3]. In addition, insomnia leads to societal costs that might include reduced productivity and more sick leave from work [2,4]. A review of the literature showed that about 9% to 15% of the western adult population suffers from insomnia symptoms and the daytime consequences thereof [5].

Although the consequences of insomnia may be severe and prevalence is substantial, only a few people seek treatment [6-8]. When help is sought, insomnia is most commonly treated with pharmacotherapy [7]. However, cognitive behavioral therapy for insomnia (CBT-I) is preferable, because CBT-I is equally effective in the short term and has more beneficial long-term effects than pharmacotherapy [9-11]. Generally, CBT-I consists of weekly sessions in which the focus lies on one or more of the following exercises: sleep restriction, stimulus control, relaxation, cognitive strategies, and sleep hygiene [12].

Although CBT-I is effective, there is a lack of knowledge and accessibility regarding this type of therapy [13]. General practitioners are often not aware of the existence of CBT-I, and neither is the general public [13]. In addition, there are too few sleep therapists to help all people with insomnia [14]. In order to increase the availability and accessibility of CBT-I, Espie et al [15] suggested a stepped model with Internet-based treatment as a first option. A meta-analysis about computerized CBT-I (CCBT-I) concluded that this therapy is a moderately effective self-help intervention for insomnia [16]. Nonetheless, adherence to insomnia and other technology-mediated treatments is often mentioned as a serious problem [17-19].

The World Health Organization (WHO) recognizes the importance of adherence to health regimes in general. They stated, “Adherence is a primary determinant of the effectiveness of treatment” [20]. In agreement with the WHO statement, Gould and Clum [21] found—in their meta-analysis of self-help treatments—that better adherence to a treatment improves the treatment effectiveness. They found that the effect size was three times higher for studies that had 75% to 100% adherence than for studies with adherence rates lower than 75%. The impact of adherence on treatment outcomes therefore warrants further investigation into how we could enhance adherence within an intervention in the context of insomnia therapy.

Various authors, for example, Beun [17] and Donkin et al [18], mention that treatment adherence is a problem for cognitive behavioral therapy (CBT) in general. Reports about adherence to various Internet-based interventions show mixed results. For example, Eysenbach [19] gives a few examples in his “law of attrition” of Internet-based interventions with adherence rates ranging from 1% to 35%. Interestingly, a meta-analysis about CCBT-I reported an average adherence rate of 78% for the six studies they included [16]. However, they did not make a distinction between treatment adherence and experimental compliance, that is, the proportion of the experimental assessments, such as questionnaires, that are completed. Thus, decisive conclusions on the exact adherence rates cannot be made.

The studies in this paper are conducted in the context of the Sleepcare project [22,23], which aims at the development of a virtual sleep coach that delivers personalized, automated sleep therapy via a mobile phone. A key challenge of this e-coach is to provide therapy support in such a way that the coachees really adhere to the regimen of the personal therapy plan. In this paper, we use the generic term coachee—instead of client, patient, user, etc—to refer to both patients and nonpatients who seek help to address their health issues. The first step in the development of a virtual sleep coach that meets this adherence challenge is the analysis of current adherence rates, current adherence-enhancing strategies, and coachees’ willingness to accept those strategies. Therefore, we conducted a meta-analysis about adherence rates in technology-mediated sleep interventions; interviewed coachees about their adherence to existing sleep-supporting technology; and discussed adherence-enhancing strategies in a to-be-developed virtual sleep coach among focus groups with potential users, sleep experts, and coaches. This complementary analysis approach provided new insights on how a virtual coach can support coachees to adhere to sleep therapy (ie, the needs and constraints).

### Study I: Meta-Analysis Adherence Rates

In order to determine whether a certain outcome is related to a treatment, adherence rates must be measured. Otherwise, it cannot be claimed that the outcome was caused by the intervention [21]. Capturing adherence data is relatively easy in technology-mediated interventions [18]. However, as there is currently no standard adherence measure [18,24,25], various measures are used. A review [18] of adherence in e-therapies found the following adherence measures: number of log-ins, completed modules, number of visits/posts to a forum, pages viewed/printed, and self-reported measures. Other measures that have been suggested are the usage time of the technology [26] and reports by a spouse or related others [24]. Different measures have different advantages and disadvantages. For example, time spent using the technology is an objective measure. However, time spent is presumably influenced by cognitive ability, reading speed, familiarity with the technology, etc [18]. Therefore, time spent does not necessarily represent treatment adherence. Moreover, there is a difference in passively using material (ie, reading, listening, watching) and actively applying this material (ie, performing the exercises) [21].

First, it is important to distinguish between at least two concepts: *treatment adherence* and *experiment compliance*. Treatment adherence refers to the extent a coachee processes and applies the content of the treatment (as provided by the coach), whereas experiment compliance refers to the coachees’ completion of the experimental assessments. Other researchers have also made this distinction. For example, Christensen and colleagues [26] respectively use the terms adherence (experience content) and dropout (research trial protocol), whereas Hebert and colleagues [27] respectively call it nonusage attrition and study attrition. Treatment adherence and experiment compliance might be related, but to our knowledge no information about this relationship has been reported in the literature.

### Study II: User Adherence to Existing Sleep-Supporting Technology

After analyzing reported adherence rates to technology-mediated sleep treatment in the literature, the next step was to study coachees’ reasons why they do or do not adhere to technology-mediated sleep interventions. To do so, interviews were conducted with people who (had) used a sleep product. The first step was to identify a sample of technology-mediated sleep products. The most familiar sleep product is probably the alarm clock. Besides alarms, there are many other sleep-supporting technologies on the market. For example, relaxation-supporting technologies, sleep-measuring apps and devices, and computerized therapies.

### Study III: Focus Group Discussions—The Envisioned Sleep Coach

A limitation of the interviews from Study II, as will be discussed in more detail in the Results section, was that they were restricted to existing products, and did not include reflections on what might technically be possible regarding adherence-enhancing strategies. During the interviews, it also proved to be difficult for participants to think of additional functionality that could improve their adherence. To address the limitations of the interviews, focus groups were organized to discuss adherence-enhancing strategies of a to-be-developed sleep coach. The aim of study III was to gain insight into coachees’ attitudes and beliefs toward these adherence-enhancing strategies, for which focus groups are particularly suited [28].

## Methods

### Study I

#### Overview

The meta-analysis was primarily performed to answer the question "How well do coachees adhere to technology-mediated insomnia interventions and diagnostic tools?*"* and, secondly, to answer the question "How does adherence relate to treatment outcome?*"* Various databases were queried—Web of Science, Scopus, PubMed, and PsychINFO—on July 8 and 14, 2014, to find studies that investigate insomnia regimes mediated by technology. The used query was: *insomnia* and *Internet-treatment*, *Internet-delivered*, *Internet-based*, *Internet-administered*, *Internet intervention*, *computerize*, *online treatment*, *Web application*, *Web-based*, *virtual*, *virtual reality*, *mass media intervention*, *smartphone*, *mobile phone*, *mobile technology*, *text message*, *handheld*, or *PDA* (personal digital assistant). In addition, the references from recent meta-analyses, and systematic reviews on self-help and computerized insomnia therapy [16,29,30] were screened for potentially relevant publications. Together, this resulted in 448 unique papers of which the abstracts were read and examined (by the first author, CH) for meeting the following exclusion criteria: no main focus on insomnia, no technology involved, treatment that does not include assignments at home, no experiment, or targeted at children. Studies on children were excluded because children’s sleep problems often differ from those of adults. Besides, children’s bedtimes are partly controlled by the parents. Therefore, interventions targeted at children have other characteristics than interventions for adults and were excluded. A total of 56 papers were read completely and the inclusion of those papers was discussed between the first and second author (CH and JL).

**Figure 1:**
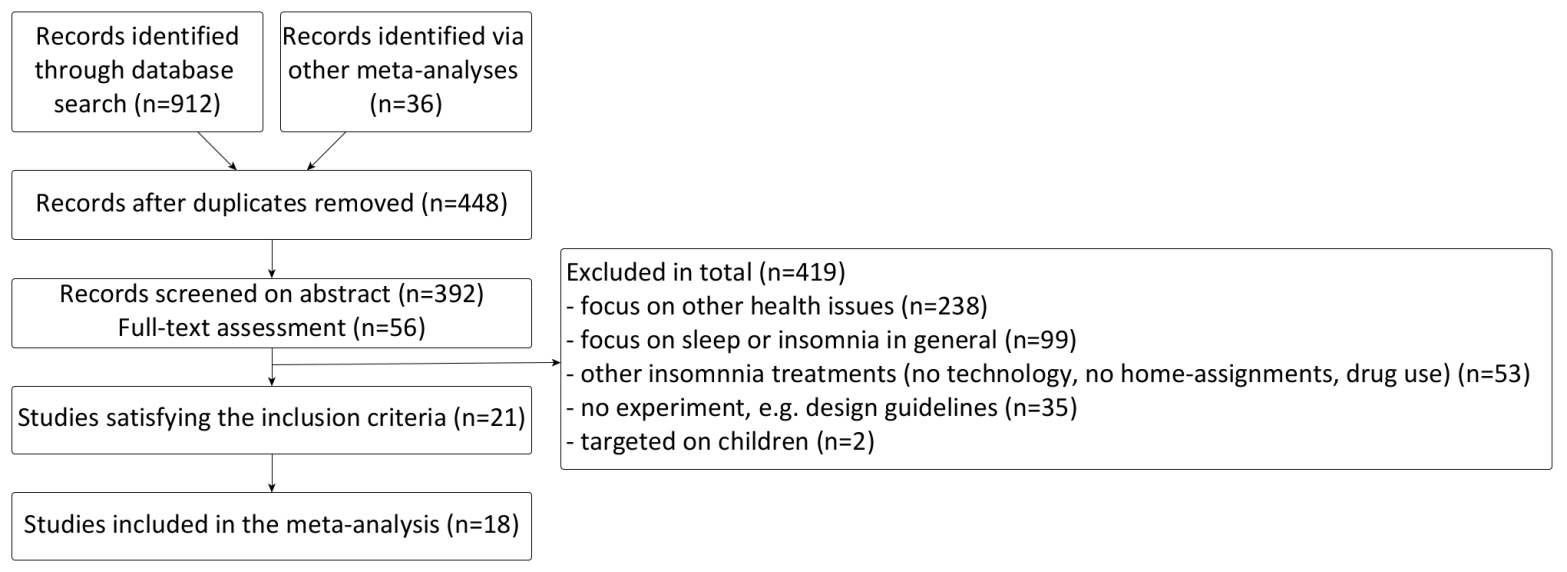
Inclusion and exclusion criteria for papers in the meta-analysis.

Figure 1 shows the flow diagram for inclusion and exclusion criteria, resulting in 21 papers from which data was retrieved. Due to a lack of reported adherence data in 3 of the papers, only 18 papers were used in the analysis. The papers selected for this meta-analysis can be found in Multimedia Appendix 1.

#### Description of Included Studies

Of the 18 included studies in this meta-analysis, 12 studies (67%) focused on CBT-I (Table 1 and Table 2). Oosterhuis and Klip [31] and Rybarczyk and colleagues [32] did include most of the CBT-I exercises in their intervention. Out of the 18 studies, 2 (11%) focused on sleep tracking by using an active sleep sampling device. The active sleep sampling device used by Riley and colleagues [33] mainly supported sleep restriction and stimulus control. The other standard CBT-I exercises were explained in an additional manual. Lawson and colleagues [34] used an active sleep sampling device inspired by Riley’s device. They developed an active sleep sampling mobile phone app which focused on sleep tracking, but did not include the other CBT-I components. Lipschitz and colleagues [35] also developed a mobile phone app, offering sleep-focused, mind-body bridging exercises. The most important assumption of mind-body bridging for sleep is that the mind needs to be rested to sleep well. Haimov and Shatil [36] studied whether providing cognitive training, such as a memory game, affects sleep.

**Table 1:** Characteristics of included studies.

First author	Condition	Number of people	Number of females/males	Mean age	Sleep problem severity, measure, score
Oosterhuis [31]	Intervention	400	63% female	55	N/A^a^
Rybarczak [32]	Intervention	14	22/16	68	PSQI^b^, 9.5
	CBT^c^	11			PSQI, 11.9
	Control	13			PSQI, 9.9
Ström [37]	Intervention	54	71/38	44	ISI^d^, 18.08
	Waiting list	55			ISI, 18.11
Suzuki [38]	Intervention	21	16/25	40	N/A
	Waiting list	22			
Ritterband [39]	Intervention	22	34/10	N/A	ISI, ≥8
	Waiting list	23			ISI, ≥8
Van Straten [40]	Intervention	126	163/84	52	72% rated SQ^e^<6/10
	Waiting list	121			68% rated SQ<6/10
Vincent [41]	Intervention	59	79/39	N/A	N/A
	Waiting list	59			
Riley [33]	Intervention 1	24	52/38	49	ISI, 8-14 (25 people)
	Intervention 2	33			ISI, 15-21 (53 people)
	SMMT^f^	33			ISI, 22-28 (12 people)
Lancee [42]	CCBT-I^g^	216	520/103	52	Sleep-50, ≥19
	CBT-I^h^	202			
	Waiting list	205			
Ritterband [43]	Intervention	14	24/4	57	ISI, 17.1
	Waiting list	14			ISI, 15.9
Espie [15]	Intervention	55	120/44	49	Met DSM-5^i^ criteria
	TAU^j^	54			Met DSM-5 criteria
	IRT^k^	55			Met DSM-5 criteria
Haimov [36]	Cognitive training (CogniFit)	34	29/22	72	Met AASM^l^ criteria
	Active control^m^	17			Met AASM criteria
Lancee [44]	Low depression	198	316/163	47	ISI, 16.73
	Mild depression	182			ISI, 18.63
	High depression	99			ISI, 20.69
					Average ISI, 18.72
Lancee [45]	With support	129	197/65	48	ISI, 16.95
	Without support	133			ISI, 17.32
Lawson [34]	Intervention	36	21/5	34	N/A
Van Straten [46]	Intervention	59	83/35	49	PSQI, 12.4
	Waiting list	59			PSQI, 11.7
Holmqvist [47]	Intervention	39	55/18	N/A	ISI, 18.72
	CBT-I	34			ISI, 18.50
Lipschitz [35]	Intervention	37	27/10	37	ISI, 7.24

^a^Not applicable (N/A)

^b^Pittsburgh Sleep Quality Index (PSQI)

^c^Cognitive behavioral therapy (CBT)

^d^Insomnia Severity Index (ISI)

^e^Sleep quality (SQ)

^f^Self-monitoring minimal treatment (SMMT)

^g^Computerized cognitive behavioral therapy for insomnia (CCBT-I)

^h^Cognitive behavioral therapy for insomnia (CBT-I)

^i^Diagnostic and Statistical Manual of Mental Disorders, 5th Edition (DSM-5)

^j^Treatment as usual (TAU)

^k^Imagery relief therapy (placebo) (IRT)

^l^American Academy of Sleep Medicine (AASM)

^m^Active control consisted of word and paint training

**Table 2:** Description of included studies.

First author	Intervention	Delivery	Treatment length	Follow-up length	Post^a^	Follow -up^b^	Adherence measure
Oosterhuis [31]	SE^c^, SH^d^, CTh^e^, RX^f^	TV^g^	8 weeks	4.5 months	Q^h^	Q	N/A^i^
Rybarczyk [32]	RX, SC^j^, SR^k^, CTh, SH	Audiotape	6 weeks	4 months	Q&D^l^	Q&D	N/A
Ström [37]	CBT-I^m^	Internet	5 weeks	9 months	Q&D	D	N/A
Suzuki [38]	CBT-I	Internet	2 weeks	3 weeks	Q	Q	N/A
Ritterband [39]	CBT-I	Internet	9 weeks	6 months	Q&D	Q	N/A
Van Straten [40]	CBT-I	TV	6 weeks	None	Q&D	N/A	Self-report
Vincent [41]	CBT-I	Internet	5 weeks	4 weeks	Q&D	Q&D	Self-report
Riley [33]	ASS^n^/CBT-I	Device	6 weeks	6 weeks	Q&D	Q&D	N/A
Lancee [42]	CBT-I	Internet	6 weeks	4 weeks		Q&D	Self-report
Ritterband [43]	CBT-I	Internet	6-9 weeks	None	Q&D	N/A	Log
Espie [15]	CBT-I	Internet	6 weeks	8 weeks	N/A	Q&D	Log
Haimov [36]	CTr^o^	PC^p^	8 weeks	None	Q&D	N/A	N/A
Lancee [44]	CBT-I	Internet	6 weeks	4 weeks	N/A	Q&D	Self-report
Lancee [45]	CBT-I	Internet	6 weeks	6 months	Q&D	Q&D	Log
Lawson [34]	ASS	App^q^	7 days	None	Q	N/A	Log
Van Straten [46]	CBT-I	Internet	6 weeks	3 months	Q&D	Q&D	Log
Holmqvist [47]	CBT-I	Internet	6 weeks	8 weeks	Q&D	Q&D	N/A
Lipschitz [35]	MBB^r^	Internet	3 days	1 week	Q	Q	Self-report

^a^Postintervention measurement instrument

^b^Follow-up measurement instrument

^c^Sleep education (SE)

^d^Sleep hygiene (SH)

^e^Cognitive therapy (CTh)

^f^Relaxation (RX)

^g^Television (TV)

^h^Questionnaire (Q)

^i^Not applicable (N/A)

^j^Stimulus control (SC)

^k^Sleep restriction (SR)

^l^Sleep diary (D)

^m^Cognitive behavioural therapy for insomnia (CBT-I)

^n^Active sleep sampling (ASS) device

^o^Cognitive training (CTr) (CogniFit)

^p^Personal computer (PC)

^q^Mobile phone app (app)

^r^Mind-body bridging (MBB)

###  Study II

#### Participant Selection

**Figure 2:**
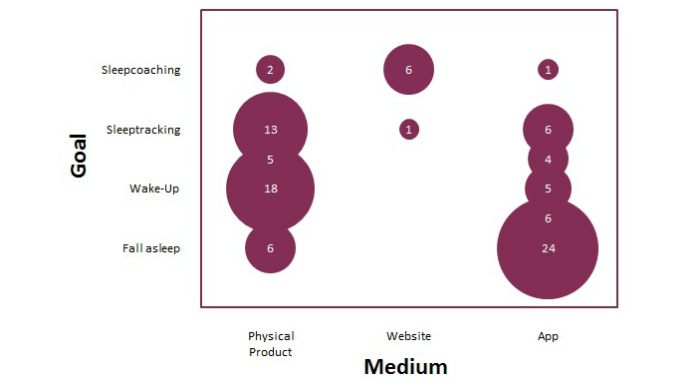
A graph showing the relationship between the goal of sleep products and the medium used. The size of each bubble indicates how many products of the 97 identified sleep products belong to that category.

In order to establish a purposive sample of users across sleep products, various sleep products were categorized. Based on their background knowledge and a media scan, the authors generated a list of 54 technologies over the course of a few months. This composed list was supplemented with apps because the goal of the Sleepcare project is to design a virtual sleep coach on a mobile phone. The first 25 Android apps and 25 iPhone apps found in Google Play and the iTunes store with the search word "sleep" on November 19, 2012, were added to the product list. A total of 7 apps were unrelated to sleep—3 games, 2 hypnosis apps, 1 unlock, 1 music timer—and were therefore discarded, resulting in a list of 97 sleep products. The categorization made in this paper aims to be simple and objective. Sleep products were categorized based on their goal and the medium used. Figure 2 shows the distribution of the products across the two dimensions: goal and medium. The size of the bubbles shows how many products belong to the intersections of the categories.

After identifying the categories of existing sleep products, the next step was to learn more about the users’ usage and adherence to the sleep products. Interviews were conducted with people who used a sleep product in each of the largest product-medium combinations (eg, apps that help people fall asleep).

#### Participants

People registered as participants at the Sleepcare project website [48] were invited to participate in the interviews if they had ever used a technology-mediated sleep product. In addition, two sleep therapists were asked to invite people who used sleep coaching products, as none of the respondents to the call used a sleep coaching product. A total of 15 Dutch persons agreed to be interviewed—6 (40%) females and 9 (60%) males—their ages ranging from 22 to 65 years (mean 37.5, SD 14.8). The mean Pittsburgh Sleep Quality Index (PSQI) [49] score was 8.0 (SD 4.0), with 12 out of the 15 (80%) interviewees having a score above 5, which is the threshold for poor sleep quality classification.

#### Interviews

Besides adherence, the interviews covered other topics to gain insight regarding users’ experiences with sleep products. Therefore, the semistructured interviews included both adherence-related questions and questions regarding the factors of the Unified Theory of Acceptance and Use of Technology (UTAUT) model [50]. The described results of the interviews in this paper, however, will only include adherence-related topics. The interviews were conducted in person, by Skype, or by telephone by the first author. The audio of the interviews was recorded. The study was approved by the Human Research Ethics Committee of Delft University of Technology.

#### Analyses

The first author (CH) performed the data analysis following the phases of thematic analysis as described by Braun and Clarke [51]. The first author familiarized herself with the data (phase 1) by conducting and transcribing the interviews, and reading the transcripts. While reading, the initial codes were generated bottom-up (phase 2). The first author coded the transcriptions and iteratively generated hierarchical codes and themes (phase 3). Short summaries of the codes and themes related to adherence were written down. The first and last author (CH and WB, respectively) discussed these summaries (phase 4) to form three final adherence-related categories (phase 5). In addition, an independent researcher applied the coding scheme to one of the interviews in order to minimize the threats to confirmability (known as objectivity in quantitative research). The independent coder confirmed the applicability and usefulness of the codes.

### Study III

#### Overview

The envisioned coach would use different adherence-enhancing strategies during the entire coaching process. For example, different roles (eg, motivator and educator) could be played by different virtual characters to increase the effect of the to-be-developed sleep coach (ie, split-persona effect) [52]. Around 25 strategies were allocated to the coach ranging from strategies involving others (eg, peers or family members), helping with planning (eg, setting goals and making commitments), and gaming strategies (eg, earning points and taking a quiz). These adherence-enhancing strategies were scripted explicitly in the scenarios in order to discuss them in the focus groups.

#### Materials

A total of 12 scenarios and 72 claims (see Multimedia Appendices 2 and 3) were written to evaluate the adherence-enhancing strategies. Scenarios consisted of stories about people and their activities, goals, and motivations regarding a system [53]. Claims stated important design decisions (eg, about the adherence-enhancing strategies) that needed to be evaluated in the focus group. Furthermore, three fictitious people varying in age, gender, family situation, and readiness-to-change were created to act in the scenarios (see Multimedia Appendix 4 for these personas).

#### Procedure and Participants

The scenarios and claims were discussed in six focus groups to evaluate the adherence-enhancing strategies. Two groups consisted of potential users, two groups consisted of coaches, and a further two groups consisted of sleep experts. Demographics of the Dutch participants can be found in Table 3. Each session lasted 2 hours and included a general introduction, an introduction round of the participants, and approximately four animated videos that represented the different scenarios. After watching one video, the participants were asked to individually rate their agreement with the claims on a 7-point Likert scale. Subsequently, participants were asked in turn to react to the claims and discuss their ideas. The sessions were videotaped for later analysis. The study was approved by the Human Research Ethics Committee of Delft University of Technology.

#### Analyses

The analysis was an iterative process of developing codes and themes in line with thematic analysis [51]. For that, the videotapes of the sessions were transcribed and summarized by the first author (CH). During that recapitulation, several codes emerged and an initial coding scheme of 12 codes was created. The first author coded the summaries according to this scheme. Additionally, a second coder, independent of the project, coded a sample of the summaries—48 of the 86 claims (56%). The second coder suggested eight additional codes. The two coders came together to discuss the coding scheme and agreed on a new scheme of 15 codes. The coding was improved (with this new scheme) by both coders, and within the sample a Cohen’s kappa of .80 was reached. Next, the first author wrote short resumes per theme, making use of quotes.

**Table 3:** Demographics of the participants per focus group.

Focus groups	Participants, n (% female)	Age in years, mean, (SD)	Number of participants with a PSQI^a^>5, n (%)	Expertise
Potential users 1	8 (38)	35 (12)	3 (38)	N/A^b^
Potential users 2	7 (71)	48 (9)	5 (71)	N/A
Coaches 1	4 (75)	51 (8)	N/A	4 coaches (relationships, lifestyle, didactical)
Coaches 2	5 (80)	50 (6)	N/A	4 coaches (lifestyle, career), 1 psychologist
Sleep experts 1	3 (67)	50 (18)	N/A	1 psychologist, 1 therapist, 1 doctor
Sleep experts 2	4 (75)	47 (14)	N/A	3 researchers, 1 psychologist

^a^Pittsburgh Sleep Quality Index (PSQI).

^b^Not applicable (N/A).

## Results

### Study I

**Figure 3:**
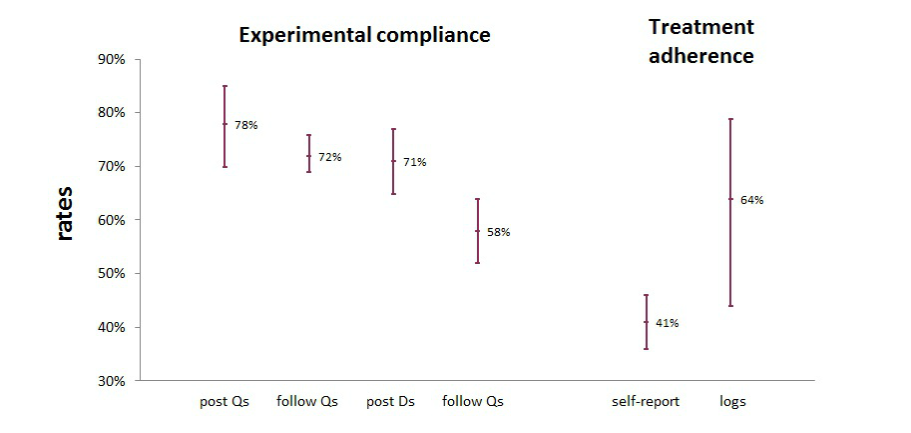
Mean compliance and adherence rates and their 95% CIs. post: posttreatment measurement; follow: follow-up measurement; Qs: questionnaires; Ds: diaries; self-report: self-reported adherence with questions; logs: automatically logged behavior.

All analyses were completed with the Comprehensive Meta-Analysis statistical package, version 3, and were based on the random-effects model. In the analyses, a distinction was made between experimental compliance and treatment adherence. All studies reported experimental compliance, and most of them (10) also reported treatment adherence (see Table 1 and Table 2). Experimental compliance was typically determined based on the completion of questionnaires and sleep diaries that were part of the study protocol; for more information see Multimedia Appendix 5. Immediately after the intervention (ie, postmeasures), the experimental compliance for questionnaires was 78% (95% CI 70%-85%), and for sleep diaries 71% (95% CI 65%-77%). At the follow-up assessments, experimental compliance to questionnaires was 72% (95% CI 69%-76%), while for diaries it was 58% (95% CI 52%-64%). These aggregated numbers are displayed in Figure 3. In Multimedia Appendix 6, individual numbers per study, aggregated rates, heterogeneity statistics, and publication bias tests can be found. Generally, the analyses indicated a substantial heterogeneity in the data, which supports the choice for a random-effects model. The shapes of the funnel plots and the Egger test did not suggest a significant publication bias.

Treatment adherence was reported in various ways, which can roughly be classified into two groups, namely self-reports and logs. Self-reports refer to questions in which participants were asked how well they adhered to the exercises. The five studies that used self-reports found that 41% (95% CI 36%-46%) of the participants met the adherence criteria set in that study. Logs refer to reports that show how many sessions were completed. A total of 5 studies used logs and found that 64% (95% CI 44%-79%) of the participants completed all sessions. If these two kinds of measures are taken together, an average treatment adherence of 52% (95% CI 43%-61%) is reached with reported adherence ranging from 28% [34] to 100% [35] across the 10 studies.

In Figure 3 the compliance and adherence rates and their 95% confidence intervals are shown; as can be seen, the self-reported treatment adherence is significantly different from the experimental compliance rates (nonoverlapping confidence intervals). Furthermore, two meta-regressions were run with studies that reported both experimental compliance and treatment adherence in order to discover a possible relationship between these two measures (experimental compliance and treatment adherence): one meta-regression with experimental compliance to postquestionnaires as the explanatory variable and logged treatment adherence as the outcome variable, and the other meta-regression with experimental compliance to follow-up questionnaires as the explanatory variable and self-reported treatment adherence as the outcome variable. These variables were chosen because most data were available for these combinations of variables. Both analyses did not reveal significant relationships between experimental compliance and treatment adherence (both had *P*>.05).

**Figure 4:**
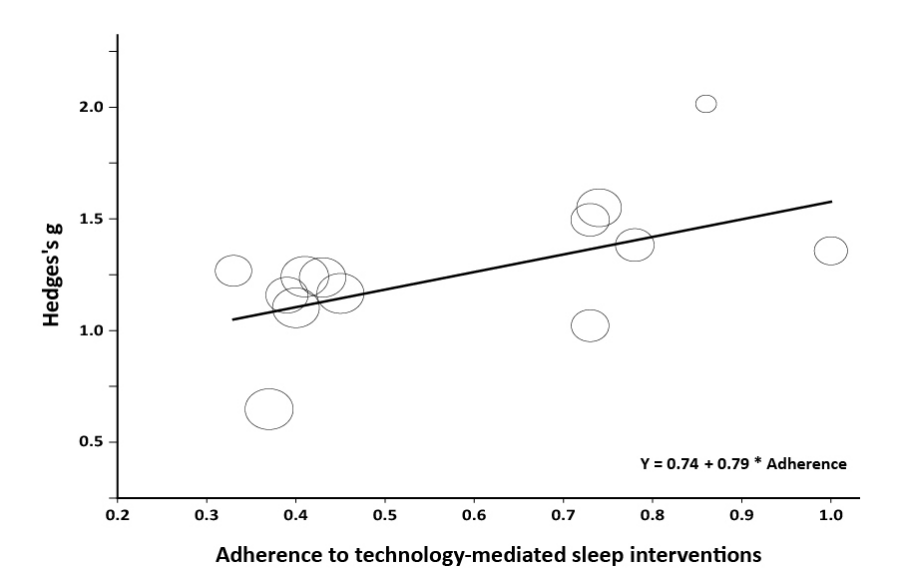
Meta-regression of adherence on effect size of treatments. The circles represent the individual studies [15-46]. The circle size indicates the weight of the study. The effect size is given in standard difference in means.

Lastly, the relationship between treatment adherence and the effect size of the individual treatments was explored. Multimedia Appendix 6, Figure 4, and Table 4 show the results of the meta-regression analysis. The analysis revealed a significant model (Q_model_=5.05*,* df=1*, P*=.03), with a coefficient of 0.79 (*Z*=2.25, *P*=.03) for adherence. In other words, treatment adherence and treatment effect are positively correlated. For example, if adherence increases with 0.30 (30%), this would coincide with a 0.24 increase in effect size (Hedge’s g) of the treatment, which is an increase of a small effect size of 0.20. The analysis also found that 75.4% (I^2^=75.4, Q=48.87, df=12) of the total variance in effect size could be explained by the variation between the studies. Of this 75.4%, 40% (R^2^=.407, T^2^_total_=.059, T^2^_unexplained_=.035) could be explained by treatment adherence.

**Table 4:** Statistics of the meta-regression of adherence and effect size of the individual treatments.

Statistics meta-regression	Coefficient	Standard error	95% CI	*Z*	*P* (2-sided)
Intercept	0.74	0.20	0.35-1.13	3.69	<.001
Adherence	0.79	0.35	0.10-1.47	2.25	.03

### Study II

#### Overview

The three main categories related to adherence are usage, effectiveness, and adherence (see Textbox 1).

**Main themes mentioned by participants in the interviews.**

Usage

- Intention

- Two reasons for usage
  - Overcome sleep problems
  - Interest in the product
- When used
  - Sleep trackers, alarms, relaxation: used in the evenings
  - Sleep coaches: varying usage times

Effectiveness

- Per product type
  - Therapy-related products: no noticeable effect
  - Alarms: ambiguous effect, wake-up is okay, but not waking up better

Adherence

- Keep using
  - Therapy-related products: personal attitude
  - Consumer products: need functionality
- Not using
  - Consumer products
    - No need for functionality (anymore)
    - Product does not work
    - Forget to use product

#### Usage

Two initial reasons for using a product emerged from the interviews. First, all interviewees used the product to overcome some of their sleeping troubles. Interviewees wanted to wake up better, initiate or maintain sleep, and/or increase insight into their sleep. Second, some interviewees used a product because they thought the product in itself was interesting. Above all, this holds for the sleep-tracking apps. Most products—alarms, automatic sleep trackers, and relaxation support—were used in the evening before going to sleep. Most of those products, however, were not used on the weekend. Sleep coach usage varied, depending on the kind of assignments included in the product (eg, diary, relaxation, sleep hygiene exercise, bedtime scheduling).

Participants' quotes regarding reasons to start using sleep products were as follows (translated from Dutch):

The reason was that in my opinion I was awake too often, and too long. I could not fall asleep anymore.

Interview #11, online sleep therapy

Friends of mine had the app and I wanted to try it as well.

Interview #4, sleep tracking app

Participants' quotes regarding usage of sleep products were as follows (translated from Dutch):

I turn it on in the evening when I am lying in bed and want to go to sleep

Interview #1, relaxation app

Every day I needed to get up I used it, but on the weekends, for example, when I don’t need to get up I didn’t do anything with the app.

Interview #7, sleep tracking app

Actually, I did it whenever it suited me [about filling in a sleep diary].

Interview #10, online sleep therapy

#### Effectiveness

One of the initial arguments for using a product was to overcome some kind of sleeping problem. However, the online sleep therapies were not perceived as having an effect on the interviewees’ sleep problems. Additionally, interviewees mentioned that it was hard to determine if the therapy improved sleep in the long term because they tried several things. Nevertheless, most interviewees took some advice that worked for them and continued applying it. Furthermore, sleep tracking apps as well as online sleep coaches provided the interviewees with more insight into their sleep and habits. Products that wake interviewees up (ie, smart alarm apps and wake-up lights) were assessed ambiguously. Both types of products did what was expected of them, namely wake the interviewee up. However, the effect of waking up better with the product was doubted. Moreover, smart alarms did not seem to fit into interviewees’ daily lives (see quote below from interview #4, sleep tracking app).

Participants' quotes regarding the effectiveness of sleep products were as follows (translated from Dutch):

The goal to sleep better was not reached

Interview #12, online sleep intervention

I have no clue if it helped, because it is going better at the moment, but I did other things in that same time period.

Interview #10, online sleep therapy

Also getting out of bed when I am awake for more than 30 minutes. The advice has helped, yes.

Interview #10, online sleep therapy

It measures the sleep debt that you are building up, that was effective.

Interview #6, sleep tracking app

It did what it supposed to do, wake me up.

Interview #7, sleep tracking app

Still not very well, but it became a little bit better, a little bit more pleasant

Interview #5, sunrise alarm

Problem of the app [smart alarm] is that you do not know what time you will wake up exactly. If I have an appointment somewhere I need an hour to get ready. If you do not know how late your alarm will go, it is hard to plan.

Interview #4, sleep tracking app

#### Adherence

In general, interviewees perceived their own usage as sufficient. Interviewees especially perceived their own personal attitude, beliefs, and willpower as important for adherence. These personal characteristics were regarded as particularly important for adherence to therapy-related products. The usage of consumer products (eg, an alarm clock) was continued, because the interviewees needed the functionality. The main arguments for not using a consumer product were (1) no perceived need for the product, (2) a perceived lack of effectiveness, and (3) the interviewee forgot to use the product.

Participants' quotes regarding the satisfaction about adherence of sleep products were as follows (translated from Dutch):

It went well. I cannot remember not doing the exercises.

Interview #13, online sleep therapy

[about doing the exercises everyday] Well, that went ok.

Interview #10, online sleep therapy

I use it 3 or 4 times a week, depending on my needs.

Interview #1, relaxation app

Participants' quotes regarding the effect of personal attitude, beliefs, and willpower on adherence of sleep products were as follows (translated from Dutch):

I tried to keep myself to it as much as possible, and of course I missed a day now and then, but I tried really hard

Interview #10, online sleep therapy

You cannot just resign and accept your sleep problem.

Interview #10, online sleep therapy

I was really motivated, so that makes a difference.

Interview #10, online sleep therapy

[What dragged you through it?] My will. I intended to do it. I started it and I wanted to get a grip on my sleep problem, so I had to follow through. [So your own determination?] Yes, without discipline you will not succeed.

Interview #11, online sleep therapy

I felt like, I started it, so I should finish it.

Interview #12, online sleep intervention

You have to be serious about it. It is a therapy that you really have to complete, otherwise it will not have an effect. So, you have to believe in it.

Interview #13, online sleep therapy

If you do not recognize the need to change, you should not start it.

Interview #13, online sleep therapy

Participants' quotes regarding reasons for using and not using sleep products were as follows (translated from Dutch):

You have to set an alarm, anyway.

Interview #6, sleep tracking app

During the holidays there is no need for an alarm.

Interview #5, sunrise alarm

I did not have the impression that the app could change my sleeping pattern.

Interview #6, sleep tracking app

I simply forgot it.

Interview #4, sleep tracking app

### Study III

#### Overview

The most obvious emerging themes in the focus groups were *users in control* and *doing it for your own sake*. In general, participants believed in the personal strengths and willpower of users to adhere to the proposed sleep coach. Furthermore, the adherence-enhancing strategies and motivation were discussed. See Textbox 2 for an overview of the results.

**Main themes mentioned in the focus groups with potential users, coaches, and sleep experts.**

Users being in control

- Control increases commitment and motivation

Doing it for own sake

- Phrase that was strongly believed in was "I do it for my own sake"

Motivation: three conflicting ideas

- If coach is downloaded, then the user is motivated
- Downloading does not imply motivated usage
- Motivation can arise while using

Adherence-enhancing strategies

- Awarding points for progress

- Not seen as appropriate for sleep coach; however, awarding points can work against own expectations
- Giving compliments
  - Not too often, not for nonsignificant actions
  - Should contain context, and vary over time
- Providing reminders
  - Should not be necessary; however, they are practical
  - Reminders are perceived as positive when set by the users
- Provide rationale: two types of people
  - Type 1: first experience exercise, then explanation
  - Type 2: first explanation, then perform exercise
- I am not the only one
  - Provide a forum, stories from others, amount of app users, statistics

#### Users in Control

Potential users, coaches, and sleep experts agreed that the users should be in control. Different arguments were given. The coaches and sleep experts mainly argued that giving the user more control increases commitment and motivation. The potential users argued that they use the sleep coach for their own sake, so they want to be in control themselves. Another argument was that not being in control could lead to irritation. Aspects that participants believed the users should be able to control were the following: reminders, amount of information given by the app, scheduling exercises, decisions about motivation level, sharing therapy progress, sharing the outcome of questionnaires, and parameters shown in sleep diary overview.

#### Doing it for Your Own Sake

The other interesting theme was *doing it for your own sake*. In one scenario, there was an example exercise which entails making a list of people who can help you. In general, this exercise met resistance by the potential users. The idea that you have to solve your problems yourself was dominant. Users would feel ashamed to ask for support, and they believed the virtual sleep coach should help them. On the other hand, the coaches stated that thinking about social resources, such as family and peers, could really help people. The coaches mentioned that coachees usually consult a coach exactly because they try to solve their problems themselves, instead of asking their social resources for help. One potential user shared that only informing other people about her sleep problems and therapy already helped her a lot, even without asking for support. Nonetheless, the general mind-set was that people use such a coach for their own sake, and that they are and should be able to take responsibility for their own adherence.

#### Motivation

The claims underlying the envisioned usage scenarios stated that users should be motivated before they start sleep treatment, otherwise the probability of dropping out would be too high. The focus groups with the sleep experts manifested three different ideas about motivation. Some of the sleep experts argued that people will be motivated at least a little bit when they have downloaded the app, since that requires some effort. On the other hand, it was also argued that someone could show interest in the sleep coach, but he or she would not necessarily be motivated to use the sleep coach. Third, it was argued that motivation could arise during different phases of a therapy; for example, after someone performs an exercise and experiences its effects. In that situation, users would not need to be highly motivated at the beginning of the therapy.

#### Adherence-Enhancing Strategies

##### Overview

Several adherence-enhancing strategies and ideas to increase motivation were scripted in the scenarios (eg, awarding points, compliments, reminders) and are discussed below.

##### Awarding Points for Progress

In general, participants reacted adversely to the idea of awarding points as described in the scenarios, mainly because the sleep coach was seen as a serious program for adults. Furthermore, it was believed that a point system is not appropriate for sleep exercises, but more for workout programs. Nevertheless, a few participants spoke up and said that they liked the idea of points. A few stories came up about how awarded points motivated participants in other domains against their own expectations. Thus, points might improve adherence, despite users’ initial reluctance.

##### Compliments

Furthermore, both the coaches and the potential users made negative remarks about the compliments. In principle, both groups thought compliments could enhance a user’s experience, but compliments should not be given too often, or for nonsignificant actions. They argued that compliments should contain context and should vary over time. Otherwise, compliments would not increase motivation.

##### Reminders

Reminders were embraced, as long as users are in control of those reminders. The users wanted to set the reminders themselves, because sometimes "you just forget to do something." On the other hand, some users stated they do not need reminders, since they are using the sleep coach for their own sake. Besides that, they argued that they are adults, are motivated, and have self-discipline. Both the coaches and the sleep experts agreed with those potential users and thought that reminders should not be necessary. However, from a practical point of view, they understood that people sometimes do forget to do therapy exercises.

##### Ideas Generated by the Participants

Other ideas to improve motivation mentioned by the participants were as follows: provide a rationale, show statistics, decrease the feeling of being alone, positive feedback, taking small steps, choosing your own coach, demanding a small investment before starting, and showing how much effort users have already invested.

According to the sleep experts, rationales for doing an exercise should be given before users start an exercise. However, the potential users and coaches mentioned there are two types of people: people who want to know how and why things are the way they are, and people who just want to experience an exercise and afterward gain an understanding of that exercise.

Secondly, different ideas were offered to ensure that users do not feel as if they are the only ones suffering from sleep problems. Ideas included a forum (suggested by users and coaches), reading stories from peers (suggested by coaches), and a measure that indicates how many people are using the app (suggested by sleep experts). The idea was that decreasing the feeling of being the only one with sleep problems could increase the motivation of users to adhere to the sleep therapy.

## Discussion

### Study I

The meta-analysis of adherence rates found a mean experimental compliance of at least 70%, except for the follow-up diaries. Filling out a diary every day for a full week a few months after the intervention requires quite some effort, which might explain a lower adherence rate (58%) to follow-up diaries than to the other experimental compliance measures. The average self-reported treatment adherence was 41%, whereas the average logged adherence was higher at 64%. This is surprising because the self-reported adherence was less "strict" than the logged adherence; for instance, users were categorized as adherent when they reported doing an exercise a certain number of times (eg, more than 4 times a week), while the logged adherence rate was based on doing all exercises. The average treatment adherence rate (logged and self-reported, combined) was 52%. Although self-reports and logs are not exactly the same, they both measure adherence and are similar enough to be combined. Nevertheless, this general adherence rate of 52% should be interpreted carefully.

Furthermore, this meta-analysis confirmed that treatment adherence is positively related to treatment effect when it comes to technology-mediated insomnia treatment. Moreover, this analysis showed that experimental compliance and treatment adherence are not related. In other words, the percentage of participants who filled out questionnaires after the intervention was not found to be an indication of how well people adhered to the treatment. Therefore, it seems important to distinguish between experimental compliance and treatment adherence.

The quality of the individual studies was not assessed using a predefined algorithm, which might be a limitation. However, the included studies were all published in peer-reviewed journals and proceedings, which warrant an acceptable level of quality. Besides, Glass and colleagues argue that all studies should be included [54]. According to them, all studies should be reviewed in context with each other regarding the topics at issue, not necessarily regarding the overall quality of each study. Since adherence is the main focus of this paper, instead of examining a possible relationship between general study quality and adherence [54], the methodological differences of measuring adherence were reviewed by differentiating between experimental compliance and treatment adherence, and self-reported and logged adherence.

### Study II

The aim of the interviews was to gain more insight into the reasons why coachees adhere to technology-mediated sleep products. Surprisingly, interviewees were quite satisfied with their own usage, which departs from the average 52% adherence rate found in the meta-analysis. The reasons why people started using a product were either out of interest or to overcome sleep problems. However, the products’ effectiveness was doubted by the interviewees and was given as a reason to stop using a product. In interviewees’ own opinions, they continued to use consumer products because they needed the functionality, whereas they adhered to therapy-related products because of their own attitudes, beliefs, and willpower. Previous research has also identified functionality as an important determinant for adherence in online sleep treatment [55]. Reasons for nonadherence were as follows: no need for the functionality, lack of effectiveness, or just forgetfulness.

Furthermore, it seemed challenging for interviewees to identify adherence-enhancing strategies in the products. It was also difficult for them to come up with an answer to the question of what could be added to the product to help them continue to use the product.

### Study III

Focus groups were organized to discuss adherence-enhancing strategies. In addition to motivation, *users in control* and the awareness to *do it for your own sake* proved to be important for adherence. The focus groups provided insights into the up- and downsides of adherence strategies, such as awarding points, compliments, reminders, and community building.

### General Discussion

#### Positive Attitudes Toward Adherence

The interviews and focus groups both revealed that people strongly believe willpower is an effective adherence strategy. Participants believed that their personal attitudes, beliefs, and motivation would ensure that they stick to their intentions of using a product. This result should be interpreted with caution because of three phenomena. First of all, sleep deprivation increases ego depletion [56]. In other words, when people are tired their willpower decreases and it will become more difficult to adhere to anything, including a virtual sleep coach. Second, the interviewees attributed their adherence to their own commitment and attitude, while nonadherence was attributed to malfunctioning of the product. This result should also be interpreted with caution because this phenomenon is in accordance with the self-serving bias. The self-serving bias states that successes are attributed to internal factors, while failure is attributed to external factors [57]. Therefore, the "good" adherence rates in the interviews were attributed to the interviewees' own willpower. Third, the participants in the focus groups were quite optimistic about their anticipated future adherence. Being optimistic about oneself and the future is one of the most robust biases (optimism bias) in psychology [58,59]. Several explanations for this unrealistic optimism has been offered, for example, ignoring everything that could go wrong [58], putting too much weight on current intentions [60], or having too much faith in willpower for future events [61]. These three phenomena provide reasons for treating participants’ optimism toward adherence with caution.

#### Aversion to Adherence-Enhancing Strategies

Apart from relying on willpower for adherence, aversion to adherence-enhancing strategies emerged during the focus groups. Therefore, when designers implement adherence-enhancing strategies they should not assume that users would initially agree with the usefulness of these strategies.

Various design principles for a virtual sleep coach can be adopted from the interviews and focus groups. The first design principle covers functionality. During the first usage phase, the sleep coach should immediately tickle users’ interest, for example, by providing automatic sleep tracking. In the interviews, it appeared that interest made coachees start using products. Next, the sleep coach can provide an already-needed functionality (eg, an alarm clock). According to the interviews, a needed functionality ensures that users keep using a product. Lastly, reminders need to be a part of the sleep coach. Reminders make sure that users do not simply forget to adhere to the coach. Both the participants in the interviews and focus groups indicated that sometimes they just forget to use a product. Participants in the focus groups showed a positive attitude toward reminders as long as the users were in control over the reminders. Therefore, including reminders in a sleep coach would be a good first step in future research to increase adherence.

A second design principle could be to withhold adherence support at the start of the intervention (ie, to postpone possible help by a virtual sleep coach). In this way, the coachees are acknowledged and respected as serious, motivated, and autonomous adults. Coachees can prove that they adhere to the assignments of the sleep coach; however, the virtual coach can detect when coachees fail to do their assignments, and then offer support. This support can take different forms (reminders, compliments, awarding points, etc) and can be varied over time based on the needs of the coachee.

A third design principle that can be applied is explaining why willpower does not guarantee success. After such an explanation, the understanding of the added value and acceptance of adherence-enhancing strategies might increase. On top of that, users could be given the control over the employment of adherence-enhancing strategies.

In the authors’ opinion, the most important overall design principle is balance. Coachees should not feel overwhelmed with adherence-enhancing strategies, but appreciate some occasional support. Personalization of the virtual sleep coach can ensure that the perfect balance is reached for each and every user. For example, some users might need and appreciate reminders for filling out a sleep diary every day, while other users are more likely to forget to do their relaxation exercises.

#### Measuring Adherence

Lastly, we want to stress that studies should measure and report treatment adherence, and make a distinction between experimental compliance and treatment adherence. It is important that future studies measure and report adherence rates, since it is only by the adherence measure that it can be established whether the treatment actually induces the observed outcome. The frequently made statement that adherence is important for the outcome of a treatment [17-19] seems to be supported by the findings of the meta-regression between effectiveness and treatment adherence. As a correlational analysis does not provide insight into the direction of a causal relationship, it remains unclear how effectiveness and adherence influence each other. Nevertheless, if coachees do not follow the treatment protocol (ie, adherence rates close to zero), the outcome could be attributed to other things outside the intervention [21], for example, to the waiting-list effect. Furthermore, it is important to make a distinction between experimental compliance and treatment adherence, since these seem to be two distinct constructs as the meta-analysis found no correlations. An earlier meta-analysis about the effectiveness of CCBT-I found a rather good "adherence rate" of 78% [16]. However, this rate would be considered as experimental compliance according to the definition used in this paper. Similar experimental compliance rates—79%, 72%, 70%, and 57%—were found by the meta-analysis, although treatment adherence was significantly lower. The average self-reported treatment adherence was 42%, whereas the logged treatment adherence was 64%. Although no significant difference between these two measures was found, it is important to consider how adherence is defined and measured. A study [62] that compared a paper diary with an electronic diary found a tremendous difference between self-reported adherence (90.5%) and logged adherence (10.9%) for a paper diary. Lastly, the question remains whether adherence in experimental settings resembles adherence in nonexperimental real-life settings. It could be that adherence rates in experiments are higher than in real-life situations. One possible explanation is the sunk-cost fallacy [63]. To illustrate, experiments demand more from participants regarding (pre-) measurements and participants might therefore be more committed to the intervention. When starting a treatment, they have already invested more time (ie, the sunk cost) compared to patients in nonexperimental settings, and are therefore less likely to drop out.

#### Research Quality

In order to review the quality of our research, it is helpful to know what we did to take care of the credibility, transferability, dependability, and confirmability of our studies [64]. Firstly, threats to all four concepts were minimalized by utilizing three different research methods—meta-analysis, interviews, and focus groups. Furthermore, the credibility of our findings is also consolidated by data source triangulation—literature, current users, potential users, coaches, and sleep experts. Additionally, honesty from our informants was reinforced by stating there are no right or wrong answers, and by allowing them the possibility to withdraw at any moment. We also had regular debriefing sessions between the executors and supervisors in order to strengthen credibility. The level of transferability to other application fields can only be judged by the readers, since they have the knowledge of these other domains [65]. Furthermore, future work can be done to replicate these findings in other fields. Transferability and dependability assessments are supported by descriptions of the research methods and Multimedia Appendices. Lastly, confirmability was addressed by audit trials and the second coders.

### Conclusions

In conclusion, treatment adherence seems important for the effectiveness of technology-mediated insomnia treatments. Individuals expect that they will adhere well to such treatments and would not gain much from adherence-enhancing strategies. They believe willpower is an effective adherence strategy. The 52% average treatment adherence reported in this paper, however, suggests that there is room for improvement. A virtual coach should be able to cope with this “adherence bias,” and persuade users to accept adherence-enhancing strategies (eg, reminders, compliments, and community building). Future research is needed to test the four derived design principles for a virtual coach, which might help to realize a substantial improvement.

## Appendix

Multimedia Appendix 1 Studies included in the meta-analysis.

Multimedia Appendix 2 Precontemplation scenario.

Multimedia Appendix 3 Claims in Dutch and English.

Multimedia Appendix 4 Short summary of personas.

Multimedia Appendix 5 General notes about the meta-analysis.

Multimedia Appendix 6 Results of the meta-analysis.

## Abbreviations

AASM: American Academy of Sleep Medicine
ASS: active sleep sampling
CBT: cognitive behavioral therapy
CBT-I: cognitive behavioral therapy for insomnia
CCBT-I: computerized cognitive behavioral therapy for insomnia
CTh: cognitive therapy
CTr: cognitive training
D: sleep diary
DSM-5: Diagnostic and Statistical Manual of Mental Disorders, 5th Edition
IRT: imagery relief therapy (placebo)
ISI: Insomnia Severity Index
MBB: mind-body bridging
N/A: not applicable
NIHC: Nationaal Initiatief Hersenen en Cognitie
NWO: Netherlands Organisation for Scientific Research
PC: personal computer
PDA: personal digital assistant
PSQI: Pittsburgh Sleep Quality Index
Q: questionnaire
RX: relaxation
SC: stimulus control
SE: sleep education
SH: sleep hygiene
SMMT: self-monitoring minimal treatment
SR: sleep restriction
SQ: sleep quality
STW: Dutch Technology Foundation
TAU: treatment as usual
TV: television
UTAUT: Unified Theory of Acceptance and Use of Technology
WHO: World Health Organization
