# Supplementary material for: Adherence to Technology-Mediated Insomnia Treatment: A Meta-Analysis, Interviews, and Focus Groups
Source: J Med Internet Res. 2015 Sep 4;17(9):e214. doi: 10.2196/jmir.4115 (PMC4642391; doi:10.2196/jmir.4115)
Supplement: Multimedia Appendix 1 [file jmir_v17i9e214_app1.pdf]

1. Studies included in the meta-analysis

Authors	Year	Title
Oosterhuis and Klip	1997	The treatment of insomnia through mass media, the results of a televised behavioural training programme
Rybarczyk et al.	2002	Efficacy of two behavioral treatment programs for comorbid geriatric insomnia
Ström, Petterson, Andersson	2004	Internet-based treatment for insomnia: a controlled evaluation
Suzuki et al.	2008	Evaluation of an internet-based self-help program for better quality of sleep among Japanese workers: a
Ritterband et al.	2009	Efficacy of an Internet-based behavioral intervention for adults with insomnia
Van Straten et al.	2009	Self-help treatment for insomnia through television and book: a randomized trial
Vincent and Lewycky	2009	Logging on for better sleep: RCT of the effectiveness of online treatment for insomnia
Riley, Mihm, Behar, Morin	2010	A computer device to deliver behavioural interventions for insomnia
Lancee et al.	2011	Internet-delivered or mailed self-help treatment for insomnia? A randomized waiting-list controlled trial
Ritterband et al.	2011	Initial evaluation of an internet intervention to improve the sleep of cancer survivors with insomnia
Espie et al.	2012	A randomized, placebo-controlled trial of online cognitive behavioural therapy for insomnia disorder delivered via
Haimov and Shatil	2013	Cognitive Training Improves Sleep Quality and Cognitive Function among Older Adults with Insomnia
Lancee et al.	2013	Baseline depression levels do not affect efficacy of cognitive-behavioral self-help treatment for insomnia
Lancee et al.	2013	Motivational support provided via email improves the effectiveness of internet-delivered self-help treatment for
Lawson et al.	2013	Validating a mobile phone application for the everyday, unobtrusive, objective measurement of sleep
Van Straten et al.	2013	Guided Internet-delivered cognitive behavioural treatment for insomnia: a randomized trial
Holmqvist, Vincent, Walsh	2014	Web- vs telehealth-based delivery of cognitive behavioral therapy for insomnia: a randomized controlled trial
Lipschitz, Landward,	2014	An exploratory study of an online mind-body program for poor sleepers in a community sample

Not included because of a lack of data:

Authors	Year	Title
Morawetz et al.	1989	Behavioral self-help treatment for insomnia: A controlled evaluation
Riedel et al.	1995	Sleep compression and sleep education for older insomniacs: Self-help versus therapist guidance
Chen et al.	2013	Enhancing adherence to cognitive behavioral therapy for insomnia through machine and social persuasion
