# Supplementary material for: Adherence to Technology-Mediated Insomnia Treatment: A Meta-Analysis, Interviews, and Focus Groups
Source: J Med Internet Res. 2015 Sep 4;17(9):e214. doi: 10.2196/jmir.4115 (PMC4642391; doi:10.2196/jmir.4115)
Supplement: Multimedia Appendix 2 [file jmir_v17i9e214_app2.pdf]

1. Precontemplation scenario

Precontemplation scenario

Leo downloaded the app yesterday. It appeared that the app suits his problems, so the program could help him, provided that he adhere to the exercise. During his breakfast Leo grabs his phone and he sees that he has a message from his new Sleep Coach App. He wonders what the app has to say. The coach wishes him good morning and indicates that there is a questionnaire for Leo before the sleep exercises start. Leo fills out the readiness to change questionnaire. It appears that Leo is in the precontemplation phase. The coach explains that motivation for change is an important success factor. Low motivation often leads to the non-completion of the therapy. From the questionnaire it seems that it might be inconvenient to immediately start with the sleep exercises. First, Leo and the coach will do some exercises to determine what Leo actually wants.

(precontemplation assignment 1)
The coach invites Leo to watch a movie of other people with sleeping problems. The coach shows a movie of peers who discuss their habits when they had sleeping problems. The peers were unaware of the fact that their old lifestyle have had a negative impact on their sleep quality.

(readiness to change measurement)
When the movie is finished the coach asks Leo whether he is willing to change his habits. Leo replies that he wants to sleep better, but he does not want to change his whole lifestyle. Because of his answer Leo gets another exercise belonging to the precontemplation stage. Leo cannot continue with sleep exercises, he is forced to do the motivational exercises first. Leo finishes his breakfast and he does not feel like performing another exercise. So, he closes the app, brushes his teeth and leaves for work.

Claims belonging to this scenario

1. At the start of the program, some users can be insufficiently motivated to complete the therapy.
2. Users who are not yet in the preparation phase, have a very high chance of dropping out.
3. Instead of convincing a user to change, the coach should support the thinking process about changing.

4. The user is only allowed to continue to the next readiness-to-change-phase, when the current phase is sufficiently completed.

5. Some users have not yet realized that their thoughts and habits are a problem for their sleep.

6. It is a good idea that the coach gives a warning that the user is not likely to complete the therapy due to a lack of motivation.
