# Supplementary material for: Adherence to Technology-Mediated Insomnia Treatment: A Meta-Analysis, Interviews, and Focus Groups
Source: J Med Internet Res. 2015 Sep 4;17(9):e214. doi: 10.2196/jmir.4115 (PMC4642391; doi:10.2196/jmir.4115)
Supplement: Multimedia Appendix 3 [file jmir_v17i9e214_app3.pdf]

1. Claims in Dutch and English

The claims discussed in different focus groups are listed below per scenario. The claims that are striked through were not discussed due to time limitations.

English	***Dutch***

Scenario A: introduction with Leo

<u>Potential users</u>

I want to choose immediately between different coaches, this should not be done via settings.	Ik zou meteen willen kunnen kiezen tussen verschillende coaches, dit zou niet via de instellingen moeten gaan.
Different roles (such as the coach and professor) can best be performed by different virtual people.	Ik denk dat verschillende rollen (zoals de coach en de professor) het best uitgevoerd kunnen worden door verschillende virtuele personen.

<u>Coaches</u>

The choice of using different coaches ensures a better (trust) relationship between the user and the coach.	De keuze die de gebruiker kan maken tussen verschillende coaches, zorgt voor een betere (vertrouwens)band tussen de gebruiker en de coach.
Different roles (such as the coach and professor) can best be performed by different virtual people.	Verschillende rollen (zoals de coach en de professor) kunnen het best uitgevoerd worden door verschillende virtuele personen.

English	*Dutch*

Scenario B: introduction with Marie

Sleep experts

An app which offers CBT-I is a good first step in a stepped-care model.	Een app die CBT-I aanbiedt is een goede eerste stap in een stepped-care model.
One or more therapists, who can answer questions from users, should be connected to the app.	Er zouden één of meerdere therapeuten met de app verbonden moeten zijn, die vragen van gebruikers kunnen beantwoorden.

Coaches

Splitting the therapy in small parts is a good idea.	Het opdelen van de therapie in kleine blokjes is een goed idee.
The interaction between coach and user (e.g. answering a question) improves the involvement of the user.	De interactie tussen de coach en de gebruiker (bijv. invullen/beantwoorden van een vraag) verhoogt de betrokkenheid van de gebruiker.
In the beginning, people are not inclined to give much information about themselves. The more users work with the system, the more willing they are to give information about themselves.	In het begin zijn mensen nog niet geneigd om veel informatie over zichzelf te geven. Hoe langer gebruikers met het systeem werken hoe meer bereid ze zijn om deze informatie over zichzelf te geven.

Personalization of the app based on user characteristics (established facts about the user, such as gender, name, family) ensures that the intervention will be used better than when there would be no personalization.	Personalisatie op basis van gebruikerskenmerken (vaststaande feiten over de gebruiker, zoals geslacht, naam, familie) van de app zorgt ervoor dat de interventie beter gebruikt wordt, dan wanneer er geen personalisatie zou plaats vinden.

English	***Dutch***

Scenario C: introduction with Klaartje

<u>Potential users</u>

English	Dutch
I think it's a good idea that I could indicate whether I would like more information or not.	Ik vind het een goed idee dat de ik zelf aan zou kunnen geven of ik nog meer informatie zou willen of niet.
To me it seems nice to know what the program does and what the coach does, shortly after the start of the program.	Het lijkt mij fijn om zo vroeg in het programma al kort te horen wat de coach doet en wat hij kan.
Getting in touch with other people who also follow the program would help me to adhere to the program longer.	In contact komen met andere mensen die het programma ook volgen, zou mij helpen om het langer vol te houden.
I think it's a good idea that the coach indicates how people around me could help me with certain assignments.	Ik vind het een goed idee dat de coach bij sommige opdrachten aangeeft hoe de mensen in mijn omgeving me zouden kunnen helpen bij opdrachten.

English	***Dutch***

Scenario D: introduction new lifestyle with Marie

<u>Potential users</u>

Realistic expectations are raised by the explanation, therefore it is easier to complete the program.	Door de uitleg worden realistische verwachtingen geschept die er voor zorgen dat het gemakkelijker voor mij is het programma af te ronden.
Asking a question about the information given by the app would make me remember the information better.	Het stellen van een vraag over de informatie die de app heeft gegeven, zou er voor zorgen dat ik de informatie beter onthoud.
Reading other users' stories could help me to continue using the app.	Het lezen van verhalen van andere gebruikers zou mij kunnen helpen om de app uiteindelijk langer te gebruiken.
If I do not know the purpose and the underlying principles of something, I will give up faster.	Als ik het doel en de achterliggend principes van iets niet kent, dan geef ik eerder op.
Giving a reward for correctly answering a question would motivate me to read the information carefully.	Het geven van een beloning voor het goed beantwoorden van de vraag, zou er voor zorgen dat ik gemotiveerder ben om de informatie goed te lezen.
The option to read or hear certain information again doesn't need to be	De keuze om de informatie nog een keer te kunnen lezen of horen, hoeft voor mij <u>**niet**</u>

offered to me.	geboden te worden door het programma.

<u>Coaches</u>

Asking a question about the information given by the app would make users remember the information better.	Het stellen van een vraag over de informatie die de app heeft gegeven, zorgt ervoor dat gebruikers de informatie beter onthouden.
Giving a reward for answering a question right would motivate people to read the information more carefully and answer the question correctly.	Het geven van een beloning voor het goed beantwoorden van de vraag, zorgt ervoor dat mensen gemotiveerd zijn de vragen goed te beantwoorden en de informatie goed te lezen.
When people do not know the purpose and the underlying principles of something, they will give up faster.	Als je het doel en de achterliggend principes van iets niet kent, dan geef je eerder op.
Reading stories from peers can help users with their problems.	Het lezen van verhalen van peers kan gebruikers helpen met hun problemen.

English	***Dutch***

Scenario E: inclusion/exclusion with Leo

<u>Sleep experts</u>

Excluding people from the program can be done later. This moment is too early.	Het onderscheid maken tussen de doelgroep en mensen die daarbuiten vallen kan later in het programma gebeuren. Dit moment is te vroeg.
The choice to read or hear certain information again doesn't need to be offered by the program.	De keuze om de informatie nog een keer te kunnen lezen of horen, hoeft niet geboden te worden door het programma.
If a questionnaire indicates that there could be a problem other than insomnia, then it is a good idea to have the option to directly schedule an appointment with a therapist.	Een mogelijkheid om direct een afspraak met een therapeut te maken als uit de vragenlijst blijkt dat er een ander probleem dan insomnie zou kunnen zijn is een goed idee.
For a therapist it is useful that the results of a questionnaire are saved, so that the therapist can assess the data.	Voor een therapeut is het handig als de uitslag van een vragenlijst wordt opgeslagen, zodat de therapeut dat kan bekijken.

English	***Dutch***

Scenario F: Precontemplation phase with Leo

Sleep experts

When starting the therapy, some people might be insufficiently motivated to finish it.	Sommige personen kunnen bij het begin van het gebruik van de app, onvoldoende gemotiveerd zijn om de therapie te doorlopen.
The user may only continue to the next "readiness-to-change-phase", when the current phase is completed. The coach decides on this.	De gebruiker mag pas verder naar de volgende “bereidheid-tot-verandering-fase”, als de huidige fase voldoende is afgerond. De coach beslist dit.
It is good that the coach gives a warning if it is likely that the user will not complete the therapy due to a lack of motivation.	Het is goed dat de coach een waarschuwing geeft dat de kans groot is dat de gebruiker de therapie waarschijnlijk niet zal afronden door een gebrek aan motivatie.
~~Instead of convincing a user, the coach should support the thinking processes of the user.~~	~~In plaats van overtuigen moet de coach het denkproces omtrent de bereidheid tot verandering van een gebruiker ondersteunen.~~

Coaches

It is good that the coach gives a warning if it is likely that the user will not complete	Het is goed dat de coach een waarschuwing geeft dat de kans groot is

the therapy due to a lack of motivation.	dat de gebruiker de therapie waarschijnlijk niet zal afronden door een gebrek aan motivatie.
Users who are still in the preparation phase have a high chance of quitting the program early.	Gebruikers die nog voor de preparation fase zitten, hebben een hele hoge kans om vroegtijdig met het programma te stoppen.
It is a good idea that if the user is not sufficiently motivated, the user will be offered a different but equal exercise, in order to increase the level of motivation.	Het is een goed idee dat als de gebruiker nog onvoldoende gemotiveerd is er een gelijkwaardige, maar andere oefening wordt aangeboden, om de gebruiker alsnog op het gewenste motivatieniveau te krijgen.

English	***Dutch***

Scenario G: Contemplation phase with Klaartje

Potential users

I think it is unnecessary that the coach indicates in advance approximately how long an exercise will take.	Ik vind het overbodig dat de coach van te voren aangeeft hoe lang een opdracht ongeveer gaat duren.
Giving compliments would motivate me to continue to do the exercises.	Het geven van complimenten, zou mij motiveren om door te gaan de opdrachten uit te voeren.
I would like to have the possibility to not know something, this would give me a feeling of safety.	Ik zou het fijn vinden dat de mogelijkheid om iets niet weten gegeven wordt door de slaapcoach, hierdoor zou ik me veilig en serieus genomen voelen.
If I can choose the reminder time myself, I would respond better to that notification.	Als ik de tijd van een herinnering zelf kan kiezen, dan zou ik beter op die herinnering reageren.
I think that if I am not able to do the exercise right now, it's a good idea that the coach would help me with planning an assignment.	Ik vind het een goed idee dat de coach mij zou helpen met het plannen van een opdracht, als ik deze niet op dit moment zou kunnen uitvoeren.
I would like it if the app sends me reminders for exercises, etc.	Ik zou het fijn vinden als de app herinneringen stuurt voor het uitvoeren van opdrachten etc.

A user forum would not provide me support for doing the exercises.

Een forum met andere gebruikers zou mij **<u>geen</u>** ondersteuning bieden bij de opdrachten.

<u>Coaches</u>

A user forum would support people with doing the exercises.

Een forum met andere gebruikers biedt goede ondersteuning bij de opdrachten.

Giving compliments motivates users to continue with the exercises.

Het geven van complimenten motiveert de gebruiker om door te gaan met de opdrachten.

By showing how far users are with an assignment (progress indicator), they are more motivated to continue.

Door te laten zien hoe ver gebruikers al zijn met een opdracht (voortgangsindicatie), zijn ze meer gemotiveerd om door te gaan.

It would give users a feeling of safety and being taken seriously, when the possibility to not know something is given.

Door gebruikers de mogelijkheid te geven dat ze iets niet weten, voelen ze zich veilig en serieus genomen.

English	***Dutch***

Scenario H: preparation phase with Marie

<u>Potential users</u>

I would like it if the app sends me reminders for exercises, etc.	Ik zou het fijn vinden als de app herinneringen stuurt voor het uitvoeren van opdrachten etc.
I would not mind if the coach measures my motivation level and decides (on my behalf) if I am motivated enough to go on to the next "readiness-to-change-phase".	Ik zou het <u>**niet**</u> erg vinden dat de coach mijn motivatieniveau meet en besluit (in plaats van ik zelf) of ik genoeg gemotiveerd ben om door te gaan naar de volgende “bereidheid-tot-verandering-fase”.
Giving examples would be superfluous for me. I would not use the examples to do the exercises in a better way.	Het geven van voorbeelden zou voor mij overbodig zijn. Ik zou de voorbeelden niet gebruiken om de opdrachten beter uit te voeren.
Making a list of people around me who could help me with the intended changes would help me in the process of change.	Een lijstje maken van mensen in mijn omgeving die me zouden kunnen helpen met de beoogde verandering zou verandering voor mij vergemakkelijken.

<u>Coaches</u>

If a user has not used the app for a long time, the app will send a reminder.	Als een gebruiker de app lang niet gebruikt, dan stuurt de app een herinnering.

By giving examples, users have a better idea of what to do and can therefore do the exercises in a better way.

Door het geven van voorbeelden, hebben gebruikers een beter idee van wat ze moeten doen en kunnen daardoor de opdrachten beter uitvoeren.

It is a good idea to make people think about how other people can help them with their intended change.

Het is goed om mensen na te laten denken over hoe anderen mensen hun kunnen helpen met de verandering die ze willen ondergaan.

It is better that the coach measures the motivation of the user and decides whether the user is motivated enough to continue to the next "readiness-to-change-phase", than when the users decide this for themselves.

Het is beter dat de coach het motivatieniveau meet en besluit of de gebruiker genoeg gemotiveerd is om door te gaan naar de volgende “bereidheid-tot-verandering-fase”, dan dat de gebruiker dit zelf mag beslissen.

English	***Dutch***

Scenario I: filling out the sleep diary with Leo

Potential users

Immediately starting with filling in the sleep diary (at the beginning of the use of the app) would increase my motivation to keep using the program.	Meteen starten (bij het begin van het gebruik van de app) met het invullen van het dagboek zou mijn motivatie om door te gaan met het programma verhogen.
To me it seems like it would be nice to get a reminder later in the day, when I have not completed the sleep diary yet.	Het lijkt mij prettig om later op de dag een herinnering te krijgen als ik het dagboek nog niet heb ingevuld.
Getting points would result in me doing the exercises better.	Het krijgen van punten zou er voor zorgen dat ik de opdrachten beter uitvoer.
Personalization of the app based on my preferences (such as type of coach, preferred reminder type) would make me use the app better, than if there was no personalization.	Personalisatie van de app op basis van mijn voorkeuren (zoals type coach, voorkeur voor remindertype) zou er voor zorgen dat ik de app beter zou gebruiken, dan wanneer er geen personalisatie zou plaats vinden.

Sleep experts

Immediately starting with filling in the sleep diary (at the beginning of the use of the app), would increase users' motivation to keep using the program.	Meteen starten (bij het begin van het gebruik van de app) met het invullen van het dagboek verhoogd de motivatie van gebruikers om door te gaan met het programma.

It is a good idea to send a reminder later in the day, when a user did not complete the sleep diary yet.	Het is een goed idee dat de gebruiker later op de dag een herinnering krijgt als diegene het dagboek nog niet heeft ingevuld.
It is better not give feedback to the users about their sleep during the first week. This means the user can not see any graphs about their sleep.	Het is beter om gebruikers de eerste week geen terugkoppeling te geven over hun slaap. Dit betekent dat de gebruiker geen grafiekjes en overzichtjes kunnen zien over hun slaap.
It is diagnostically important to fill in the diary immediately after someone wakes up.	Het is diagnostisch belangrijk om het dagboek in te vullen meteen nadat men wakker wordt.

Coaches

Getting points makes people do the exercises better.	Het krijgen van punten zorgt ervoor dat mensen de opdrachten beter uitvoeren.
On the basis of how well users fill in the diary, the willingness to change can be established.	Aan de hand van hoe goed gebruikers het dagboek invullen, kan de bereidheid tot verandering gemeten worden.
Personalization of the app based on user preferences (such as type of coach, preferred reminder type) would improve the app usage, compared to no personalization.	Personalisatie op basis van gebruikersvoorkeuren (zoals type coach, voorkeur voor herinneringstype) van de app zorgt ervoor dat de interventie beter gebruikt wordt, dan wanneer er geen personalisatie zou plaats vinden.

English	***Dutch***

Scenario J: Setting goals with Marie

<u>Sleep experts</u>

It is a good idea that users can specify whether they want more information or not.	Het is een goed idee dat de gebruikers zelf aan kunnen geven of ze nog meer informatie willen of niet.
Not filling in the diary for one day is not a problem.	Één dag het dagboek niet invullen is geen probleem.
Letting the program calculate the sleep efficiency is a good idea.	Slaapefficiëntie laten uitrekenen door het programma is een goed idee
It is better to first fill in the diary and set goals for yourself afterwards than vice versa.	Het is beter om eerst het dagboek in te vullen en daarna doelen voor je zelf te stellen i.p.v andersom.

<u>Coaches</u>

It is better to first fill in the diary and set goals for yourself afterwards than vice versa.	Het is beter om eerst het dagboek in te vullen en daarna doelen voor je zelf te stellen i.p.v andersom.
It is okay when some goals are determined by the sleep coach.	Het is goed als sommige doelen door de slaapcoach worden bepaald.
A goal should be realistic and measurable (i.e. something with numbers).	Een einddoel moet realistisch en meetbaar zijn (dus iets met getallen).

English	***Dutch***

Scenario K: The plan of change with Klaartje

Sleep experts

The user does not have the knowledge to develop an effective change plan, therefore it is better that the coach gives a suggestion.	De gebruiker heeft niet de kennis om een effectief veranderplan op te stellen, daarom is het beter als de coach een suggestie geeft.
It must be possible for the user to adapt the treatment plan.	Het moet voor de gebruiker wel mogelijk zijn om zelf het behandelplan aan te passen.
For the user to be in control, and therefore more motivated, is less important than a good therapy. In other words, a user can better adhere to half of a good therapy, than fully complete a bad therapy.	Dat de gebruiker in controle is en daardoor ook gemotiveerder is, is minder belangrijk dan een goede therapie. Anders gezegd, een gebruiker kan beter een halve goede therapie doorlopen, dan een slechte therapie volledig afronden.
Sharing the therapy progress is not okay.	Het delen van de voortgang van de therapie is niet oké.

Coaches

It must be possible for the user to adapt the treatment plan.	Het moet voor de gebruiker mogelijk zijn om zelf het behandelplan aan te passen.
It is a good idea to have the user give explicit consent (for example, by putting a	De gebruiker expliciete instemming laten geven (bijvoorbeeld door een handtekening

signature on the plan).	te laten zetten onder het plan), is een goed idee.
By sharing the plan with others, such as friends, family and other people with sleep problems, users are more likely to execute the plan.	Door het plan te delen met anderen, zoals vrienden, familie en andere mensen met slaapproblemen, zijn gebruikers eerder geneigd het plan uit te voeren.
Sharing the therapy progress is not okay.	Het delen van de voortgang van de therapie is niet oké.

English	***Dutch***

Scenario L: Therapy with Marie

Potential users

If I get a notification from the coach, I would like to have the possibility to snooze the reminder (repetition of the notification over 9 minutes), or to delete the notification completely.	Ik vind het een goed idee dat als er een melding van de coach komt, ik zou kunnen kiezen om de melding te sluimeren (herhaling over 9 min), of om de melding helemaal weg te drukken.
I think it's a good idea that when I turn the coach off, the app will still send a notification after 2 or3 hours.	Ik vind het een goed idee dat als ik de coach definitief wegdrukt, de app na 2 à 3 uur toch terug komt met de melding.
The explanation of an exercise should always be shown before the exercise starts.	Ik vind dat de uitleg van de opdracht altijd te zien zou moeten zijn voordat de opdracht begint.
I want to be able to click quickly through the information if I am already familiar with the information.	Ik zou snel door de informatie heen willen kunnen klikken als ik al bekend ben met de informatie.

Sleep experts

The successive steps, education, instruction, execution, evaluation and prevention of relapse, is the best way to offer a therapy exercise.	De opeenvolgende stappen onderwijs, instructie, uitvoering, evaluatie en voorkomen van terugval, zijn de beste manier om een therapie opdracht aan te bieden.

The users should be able to click quickly through the information if they are already familiar with the information.	De gebruikers zouden snel door de informatie heen moeten kunnen klikken als ze al bekend zijn met de informatie.
Sometimes the section 'education' can be skipped.	Soms kan het onderdeel ‘onderwijs’ overgeslagen worden.
The instruction belonging to an exercise should always be shown before the exercise starts.	De ´instructie van´ de opdracht zou altijd te zien moeten zijn voordat de opdracht begint.

Coaches

~~Monitoring motivation weekly is not a good idea.~~	~~Motivatie per week monitoren is **geen** goed idee.~~
~~The users should be able to click quickly through the information if they are already familiar with the information.~~	~~De gebruikers zouden snel door de informatie heen moeten kunnen klikken als ze al bekend zijn met de informatie.~~
