# Supplementary material for: Adherence to Technology-Mediated Insomnia Treatment: A Meta-Analysis, Interviews, and Focus Groups
Source: J Med Internet Res. 2015 Sep 4;17(9):e214. doi: 10.2196/jmir.4115 (PMC4642391; doi:10.2196/jmir.4115)
Supplement: Multimedia Appendix 4 [file jmir_v17i9e214_app4.pdf]

1. Short summary of personas

They were: *Marie*, a 33-year-old married mother of two young children, who is in preparation phase and got the app from her general practitioner; *Klaartje*, a 62-year -old widow, who is in contemplation phase and got the app from a friend; *Leo*, a 49-year-old married man, with three children studying, who is in precontemplation phase.
