# Supplementary material for: Adherence to Technology-Mediated Insomnia Treatment: A Meta-Analysis, Interviews, and Focus Groups
Source: J Med Internet Res. 2015 Sep 4;17(9):e214. doi: 10.2196/jmir.4115 (PMC4642391; doi:10.2196/jmir.4115)
Supplement: Multimedia Appendix 5 [file jmir_v17i9e214_app5.pdf]

1. General notes about the meta-analysis

- The numbers are based on the people who participated in the study, not on the number of people that filled out a particular questionnaire. E.g. if adherence in Lancee (2011) is calculated only based on the people who actually filled out the adherence questionnaire, then the adherence rate would be 89/168=53%

- If a study has more than one follow-up assessment, data of the first follow-up is used. This holds for the following studies
  - Lancee (2011) 4 weeks, 8 weeks, and 48 weeks follow-up
  - Lipschitz (2014) 1 week, 1 month, and 3 months follow-up
  - Van Straten (2013) 4 weeks, and 18 weeks follow-up

- The 19 people who adhered in Vincent (2009) is a hypothetical number, and is calculated as follows: Over several weeks adherence to different exercises was measured. The total times all the participants together could adhere to the exercises was 354 times. 116 times adherence was higher than a set threshold. These numbers are used to calculate the adherence percentage (116/354= 33%), which is then transformed into the hypothetical number 19 (0,33*59 = 19), which indicates how many of the 59 participants have adhered hypothetically. Note that, the calculated numbers are based on the people who participated in the study. If adherence is calculated only based on the people who filled out the adherence questionnaire, then the adherence rate would be 116/193= 60%

- Lancee (2013a) consists of three groups of participants. Participants that suffer from high depression, mild depression and, low depression.

- Lancee (2013b) compares CCBT-I with and without support.
