# Supplementary material for: Adherence to Technology-Mediated Insomnia Treatment: A Meta-Analysis, Interviews, and Focus Groups
Source: J Med Internet Res. 2015 Sep 4;17(9):e214. doi: 10.2196/jmir.4115 (PMC4642391; doi:10.2196/jmir.4115)
Supplement: Multimedia Appendix 6 [file jmir_v17i9e214_app6.pdf]

1. Results of meta-analysis

Experimental compliance post questionnaires

Study name	Raw data	Event rate	Lower limit	Upper limit	Relative weight
Oosterhuis (1997)	219 / 400	0.55	0.50	0.60	8.23
Rybarczyk (2002)	14 / 18	0.78	0.54	0.91	5.35
Strom (2004)	32 / 54	0.59	0.46	0.71	7.38
Suzuki (2008)	18 / 21	0.86	0.64	0.95	4.97
Ritterband (2009)	21 / 22	0.95	0.74	0.99	2.94
van Straten (2009)	96 / 126	0.76	0.68	0.83	7.78
Vincent (2009)	40 / 59	0.68	0.55	0.78	7.37
Riley (2010)	28 / 33	0.85	0.68	0.94	5.92
Ritterband (2011)	14 / 14	0.97	0.63	1.00	1.80
Haimov (2013)	34 / 59	0.58	0.45	0.70	7.46
Lancee (2013b) - With	115 / 129	0.89	0.83	0.93	7.34
Lancee (2013b) - Without	117 / 133	0.88	0.81	0.92	7.44
Lawson (2013)	31 / 36	0.86	0.71	0.94	5.94
van Straten (2013)	49 / 59	0.83	0.71	0.91	6.91
Holmqvist (2014)	26 / 39	0.67	0.51	0.80	6.96
Lipschitz (2014)	31 / 37	0.84	0.68	0.93	6.20
Random model		0.78	0.70	0.85	

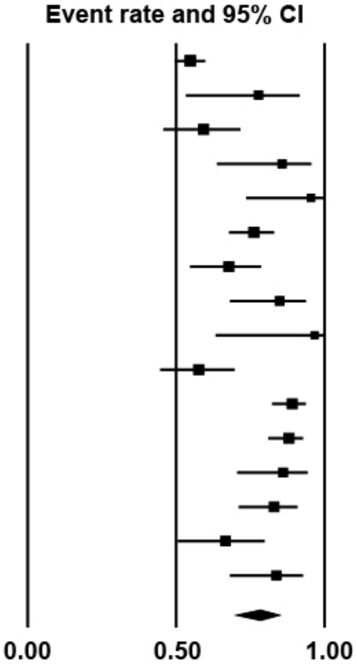

Heterogeneity: Q_{15} = 114.84, P < .001, I^2 = 86.94, indicates substantial heterogeneity in the data, which supports the choice for a random-effects model.

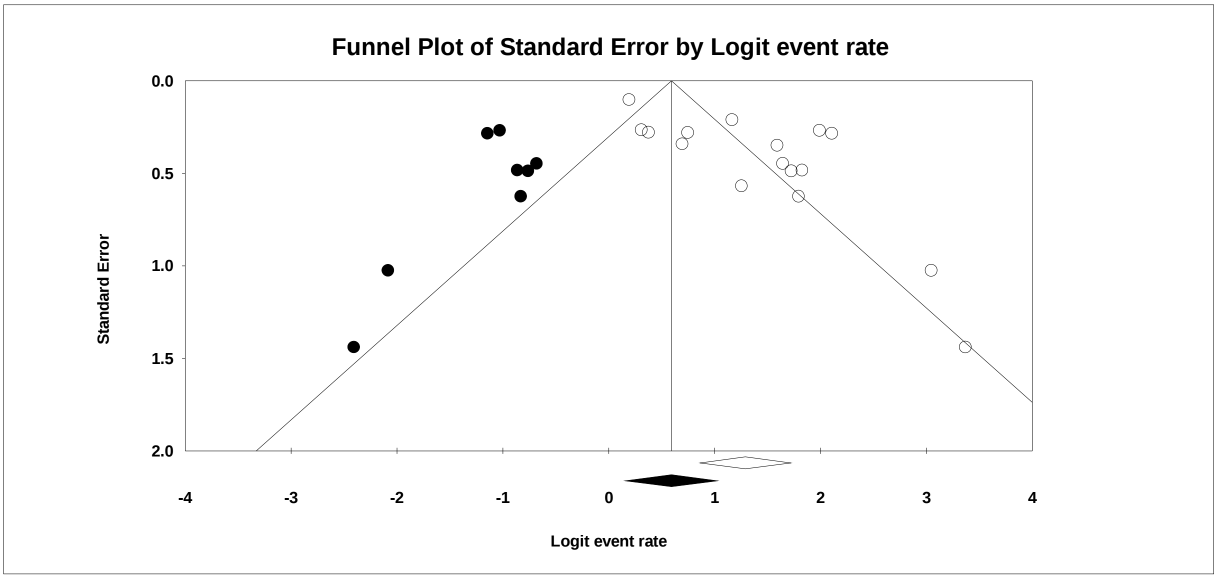

White dots indicate observed studies. The black dots indicate imputed data.

Publication bias: The funnel plot is noticeably asymmetric, with a majority of the smaller studies clustering to the right of the mean. This impression is confirmed by Egger's test (*P* = .002, two-tailed).

Experimental compliance post diaries

Study name	Raw data	Event rate	Lower limit	Upper limit	Relative weight
Rybarczyk (2002)	14 / 18	0.78	0.54	0.91	5.21
Strom (2004)	30 / 54	0.56	0.42	0.68	10.26
Ritterband (2009)	22 / 22	0.98	0.73	1.00	1.17
van Straten (2009)	100 / 126	0.79	0.71	0.86	11.46
Vincent (2009)	40 / 59	0.68	0.55	0.78	10.15
Riley (2010)	28 / 33	0.85	0.68	0.94	6.28
Ritterband (2011)	13 / 14	0.93	0.63	0.99	2.07
Haimov (2013)	34 / 59	0.58	0.45	0.70	10.49
Lancee (2013b) - With	103 / 129	0.80	0.72	0.86	11.47
Lancee (2013b) - Without	90 / 133	0.68	0.59	0.75	12.22
van Straten (2013)	37 / 59	0.63	0.50	0.74	10.36
Holmqvist (2014)	26 / 39	0.67	0.51	0.80	8.85
Random model		0.71	0.65	0.77	

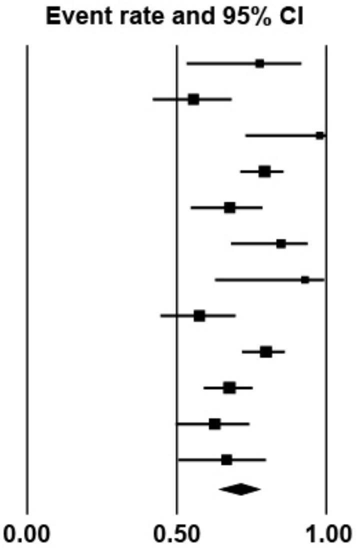

Heterogeneity: $Q_{11} = 33.26$, $P < .001$, $I^2 = 66.92$, suggests that the data is heterogeneous and supports the choice for a random-effect model.

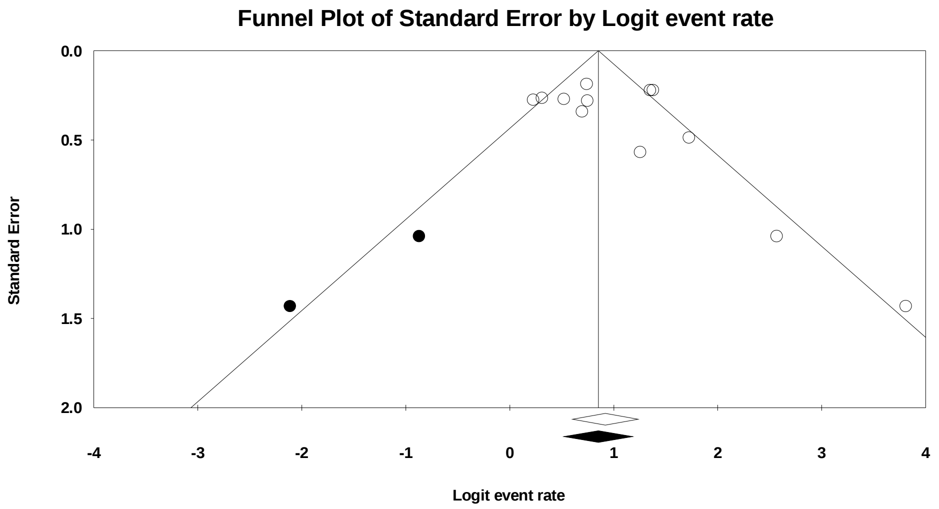

White dots indicate observed studies. The black dots indicate imputed data.

Publication bias: The shape of the funnel plot does not suggest significant publication bias, which is confirmed by Egger's test statistic, $P = 0.21$

Experimental compliance follow-up questionnaires

Study name	Raw data	Event rate	Lower limit	Upper limit	Relative weight
Oosterhuis (1997)	105 / 163	0.64	0.57	0.71	10.35
Rybarczyk (2002)	13 / 18	0.72	0.48	0.88	2.17
Suzuki (2008)	17 / 21	0.81	0.59	0.93	1.97
Ritterband (2009)	18 / 22	0.82	0.60	0.93	1.99
Vincent (2009)	35 / 59	0.59	0.46	0.71	6.25
Riley (2010)	27 / 33	0.82	0.65	0.92	2.82
Lancee (2011)	168 / 216	0.78	0.72	0.83	10.35
Espie (2012)	40 / 55	0.73	0.60	0.83	5.23
Lancee (2013a) - Low	143 / 198	0.72	0.66	0.78	10.61
Lancee (2013a) - Mild	136 / 182	0.75	0.68	0.81	10.00
Lancee (2013a) - High	71 / 99	0.72	0.62	0.80	7.68
Lancee (2013b) - With	102 / 129	0.79	0.71	0.85	7.94
Lancee (2013b) - Without	95 / 133	0.71	0.63	0.78	8.98
van Straten (2014)	43 / 59	0.73	0.60	0.83	5.47
Holmqvist (2014)	22 / 39	0.56	0.41	0.71	4.77
Lipschitz (2014)	29 / 37	0.78	0.62	0.89	3.44
Random model		0.72	0.69	0.76	

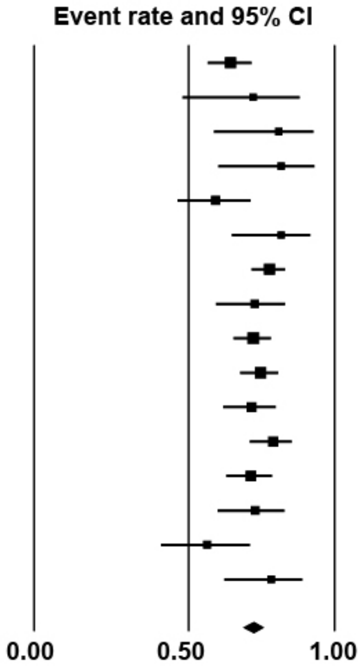

Heterogeneity: Q_{15} = 25.16, *P* = .048, I^2 = 40.39, indicates heterogeneity, which support the choice for a random-effects model.

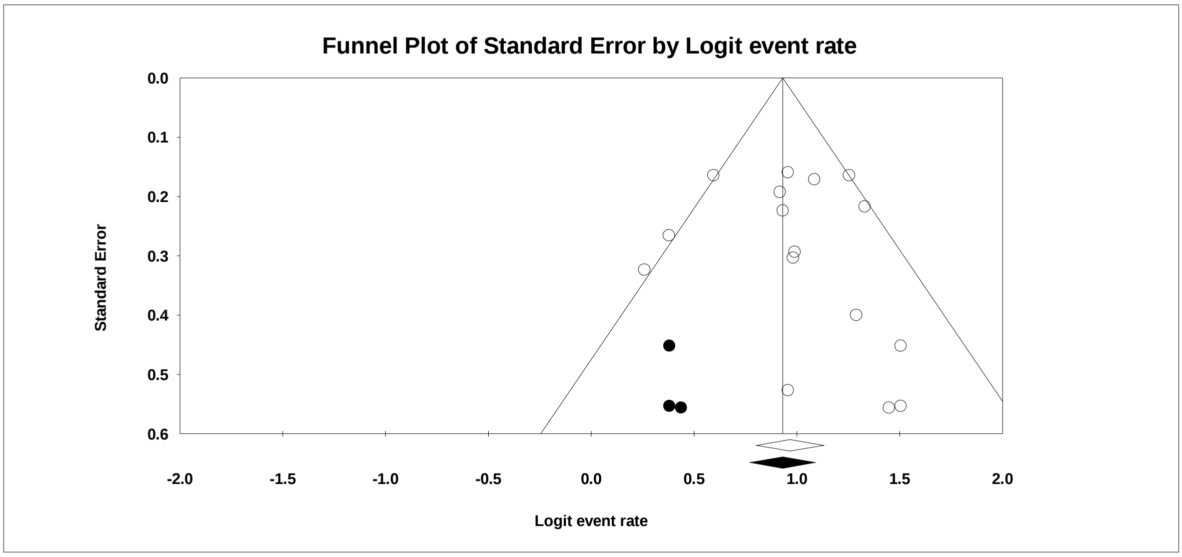

White dots indicate observed studies. The black dots indicate imputed data.

Publication bias: The shape of the funnel plot in did not reveal any indication of funnel plot asymmetry. This visual impression was also confirmed by Egger's test with *P* = 0.61, two-tailed.

Experimental compliance follow-up diaries

Study name	Raw data	Event rate	Lower limit	Upper limit	Relative weight
Rybarczyk (2002)	13 / 18	0.72	0.48	0.88	3.82
Strom (2004)	13 / 54	0.24	0.15	0.37	6.58
Vincent (2009)	35 / 59	0.59	0.46	0.71	7.54
Riley (2010)	27 / 33	0.82	0.65	0.92	4.63
Lancee (2011)	133 / 216	0.62	0.55	0.68	9.89
Espie (2012)	40 / 55	0.73	0.60	0.83	6.85
Lancee (2013a) - Low	110 / 198	0.56	0.49	0.62	9.84
Lancee (2013a) - Mild	99 / 182	0.54	0.47	0.61	9.74
Lancee (2013a) - High	47 / 99	0.47	0.38	0.57	8.76
Lancee (2013b) - With	89 / 129	0.69	0.61	0.76	8.97
Lancee (2013b) - Without	76 / 133	0.57	0.49	0.65	9.26
van Straten (2013)	29 / 59	0.49	0.37	0.62	7.62
Holmqvist (2014)	22 / 39	0.56	0.41	0.71	6.50
Random model		0.58	0.52	0.64	

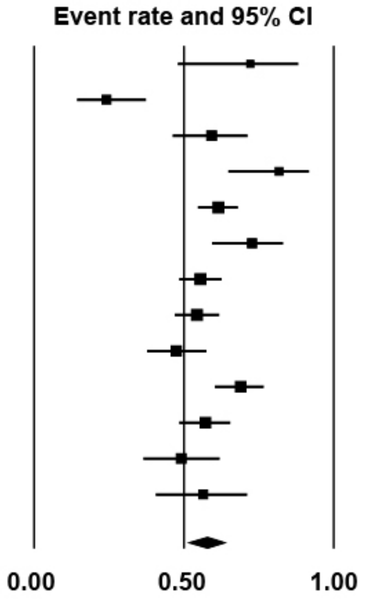

Heterogeneity: Q_{12} = 49.54, P < .001, I^2 = 75.77, indicates substantial heterogeneity and supports the choice for a random-effects model.

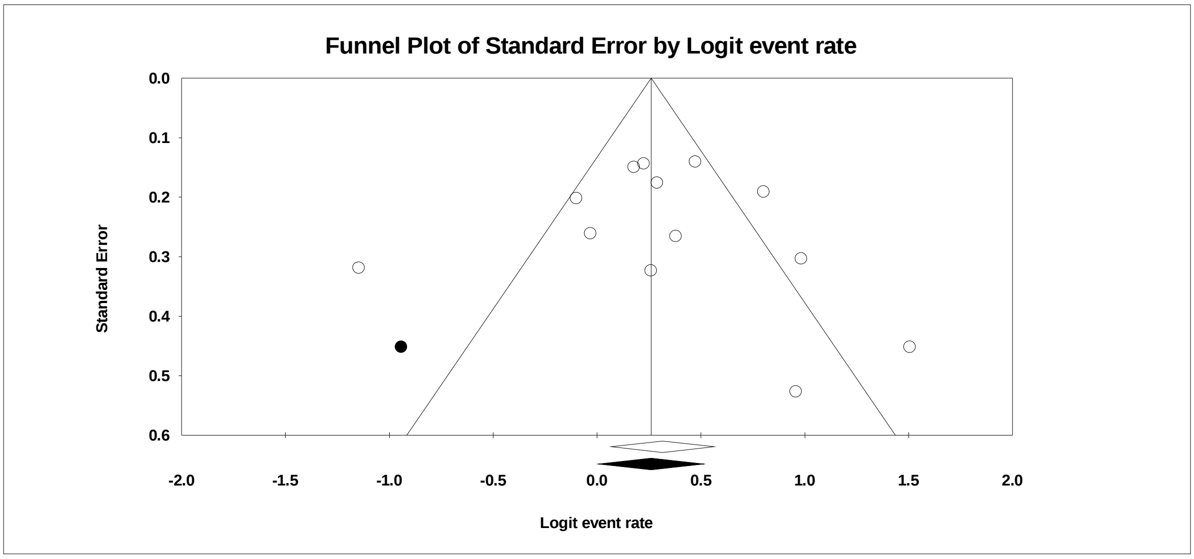

White dots indicate observed studies. The black dots indicate imputed data.

Publication bias: The shape of the funnel plot in did not reveal asymmetry. This visual impression was also confirmed by Egger's test with P = 0.75, two-tailed.

Logged treatment adherence

Study name	Raw data	Event rate	Lower limit	Upper limit	Relative weight
Ritterband (2011)	12 / 14	0.86	0.57	0.96	11.47
Espie (2012)	43 / 55	0.78	0.65	0.87	17.19
Lancee (2013b) - With	96 / 129	0.74	0.66	0.81	18.46
Lancee (2013b) - Without	53 / 133	0.40	0.32	0.48	18.66
Lawson (2013)	10 / 36	0.28	0.16	0.44	16.64
van Straten (2013)	43 / 59	0.73	0.60	0.83	17.57
Random model		0.64	0.44	0.79	

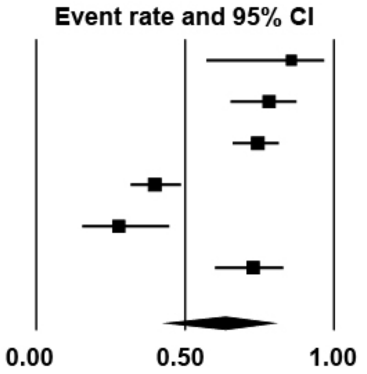

Heterogeneity: Q_5 = 59.76, *P* < .001, I^2 = 91.63, indicates that the data is heterogeneous, which supports the choice for a random-effects model.

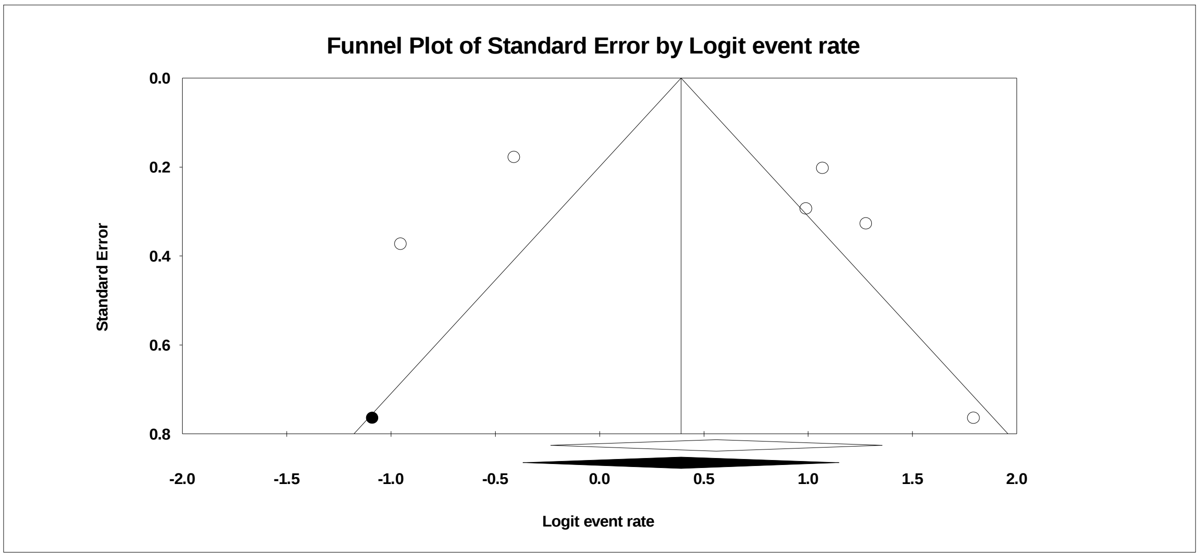

White dots indicate observed studies. The black dots indicate imputed data.

Publication bias: The shape of the funnel plot in did not reveal asymmetry. This visual impression was also confirmed by Egger's test with *P* = 0.59, two-tailed.

Self-reported treatment adherence

Study name	Raw data	Event rate	Lower limit	Upper limit	Relative weight
van Straten (2009)	47 / 126	0.37	0.29	0.46	16.16
Vincent (2009)	19 / 59	0.32	0.22	0.45	10.45
Lancee (2011)	89 / 216	0.41	0.35	0.48	19.84
Lancee (2013a) - Low	89 / 198	0.45	0.38	0.52	19.45
Lancee (2013a) - Mild	78 / 182	0.43	0.36	0.50	18.87
Lancee (2013a) - High	39 / 99	0.39	0.30	0.49	14.62
Lipschitz (2014)	29 / 29	0.98	0.78	1.00	0.62
Random model		0.41	0.36	0.46	

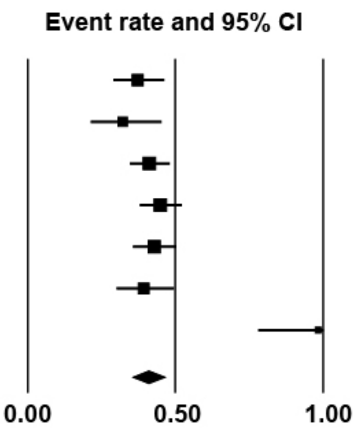

Heterogeneity: Q_5 = 13.88, P = .031, I^2 = 56.77, indicates heterogeneity and supports the choice for a random-effects model.

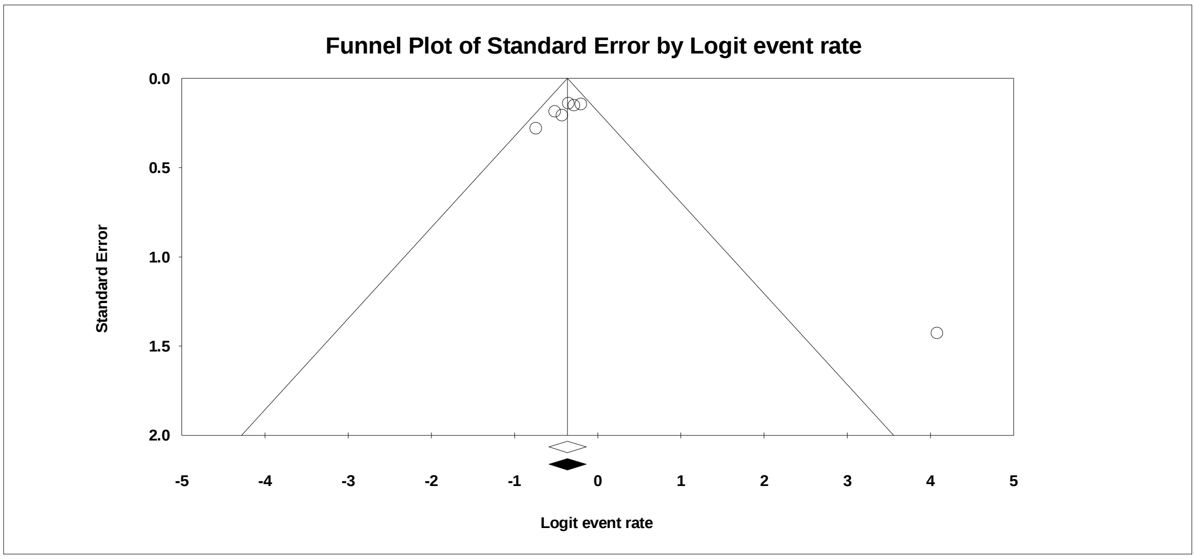

White dots indicate observed studies. The black dots indicate imputed data.

Publication bias: The shape of the funnel plot in did not reveal asymmetry. This visual impression was also confirmed by Egger's test with P = 0.29, two-tailed.
